# The Syk Kinase Promotes Mammary Epithelial Integrity and Inhibits Breast Cancer Invasion by Stabilizing the E-Cadherin/Catenin Complex

**DOI:** 10.3390/cancers11121974

**Published:** 2019-12-07

**Authors:** Toufic Kassouf, Romain Maxime Larive, Anne Morel, Serge Urbach, Nadir Bettache, Ma Cleofas Marcial Medina, Fabrice Mèrezègue, Gilles Freiss, Marion Peter, Florence Boissière-Michot, Jérôme Solassol, Philippe Montcourrier, Peter Coopman

**Affiliations:** 1 IRCM, Inserm, CNRS, Universit@#xE9; de Montpellier, ICM, 208 Rue des Apothicaires, 34298 Montpellier, France; toufic.kassouf@hotmail.com (T.K.); romain.larive@umontpellier.fr (R.M.L.); gilles.freiss@inserm.fr (G.F.); marion.peter@inserm.fr (M.P.); j-solassol@chu-montpellier.fr (J.S.); 2 CRBM, CNRS, Université de Montpellier, 1919 Route de Mende, 34293 Montpellier, France; anne.morel@crbm.cnrs.fr; 3 IBMM, Université de Montpellier, CNRS, ENSCM, 15 avenue Charles Flahault - BP 14491, 34093 Montpellier, France; nadir.bettache@umontpellier.fr; 4 Functional Proteomics Platform, IGF, Université de Montpellier, CNRS, INSERM, 141 rue de la Cardonille, 34094 Montpellier, France; serge.urbach@igf.cnrs.fr; 5 Cinvestav-IPN, Cell Biology Department, Av. IPN # 2508, Mexico City 07360, Mexico; mmarcial@cell.cinvestav.mx; 6 BioMV Department, Université de Montpellier CC25000, Place Eugène Bataillon, 34095 Montpellier, France; fabrice.merezegue@umontpellier.fr; 7 Unité de Recherche Translationnelle, ICM, 208 Rue des Apothicaires, 34298 Montpellier, France; Florence.Boissiere@icm.unicancer.fr

**Keywords:** breast cancer, tumor suppressor, protein tyrosine kinase, intercellular adhesion, tumor invasion, E-cadherin

## Abstract

While first discovered in immunoreceptor signaling, the Syk protein kinase behaves as a tumor and metastasis suppressor in epithelial cells. Its reduced expression in breast and other carcinomas is correlated with decreased survival and increased metastasis risk, but its action mechanism remains largely unknown. Using phosphoproteomics we found that Syk phosphorylated E-cadherin and α-, β-, and p120-catenins on multiple tyrosine residues that concentrate at intercellular junctions. Increased Syk expression and activation enhanced E-cadherin/catenin phosphorylation, promoting their association and complex stability. In human breast cancer cells, Syk stimulated intercellular aggregation, E-cadherin recruitment and retention at adherens junctions, and promoted epithelial integrity, whereas it inhibited cell migration and invasion. Opposite effects were obtained with Syk knockdown or non-phosphorylatable mutant E-cadherin expression. Mechanistically, Syk stimulated the interaction of the E-cadherin/catenin complex with zonula occludens proteins and the actin cytoskeleton. Conditional Syk knockout in the lactating mouse mammary gland perturbed alveologenesis and disrupted E-cadherin localization at adherens junctions, corroborating the observations in cells. Hence, Syk is involved in the maintenance of the epithelial integrity of the mammary gland via the phosphorylation and stabilization of the E-cadherin/catenin adherens junction complex, thereby inhibiting cell migration and malignant tumor invasion.

## 1. Introduction

E-cadherin/catenin-based adherens junctions (AJ) are an essential feature of epithelial cell layers. Although AJ remain stable to fulfil their adhesive role, they also exhibit plasticity [1,2,3] allowing cells to adapt to the changing environment [4]. In cancer, E-Cdh loss, mutation, or defective regulation leads to epithelial-mesenchymal transition (EMT), tumor cell invasion, and metastasis formation [5].

E-Cdh highly conserved intracellular tail associates with different cytoplasmic proteins, particularly α-, β-, and p120-catenin (Ctn) [6], and is involved in the interaction with the actin/myosin network [7,8]. E-Cdh/Ctn association, functionality, and dynamics are regulated through various mechanisms. For E-Cdh, this comprises transcriptional control [9,10,11], interaction with co-factors such as polarity proteins [3], endocytosis/recycling/degradation [12,13,14], biomechanical feedback pathways [15], glycosylation [16], and (de)phosphorylation [3,17]. Adherens junction dynamics are regulated through phosphorylation switches that can positively (stabilization) or negatively affect the complex formation and functionality [18].

The non-receptor spleen tyrosine kinase (Syk) was initially described in hematopoietic cells [19]. We and others reported that Syk is also expressed in non-immune cells [20,21]. Moreover, we demonstrated its tumor suppressor role in mammary cells [20]. Syk expression is downregulated in malignant breast cancer cells, e.g., through hypermethylation of the *SYK* promoter [22]. Clinical studies corroborated the gradual Syk loss during malignant progression of breast tumors [23,24], but also in other carcinomas and melanoma [25,26]. Syk anti-oncogenic and anti-invasive activities were demonstrated using mouse xenograft models of breast and prostate carcinoma [20,27] and melanoma [28].

The signaling pathways by which Syk exerts its anti-proliferative and anti-invasive effects in epithelial cells remain unknown, and undoubtedly differ from the ones in hematopoietic cells where Syk appears to be pro-proliferative and pro-survival [29]. It is crucial to understand the mechanisms underlying this dual role because Syk kinase inhibitors might potentiate the effect of certain chemotherapeutic drugs in vitro [30] and they are being clinically evaluated but “their use might be inappropriate for people with a family history of breast cancer” [31]. Using a quantitative SILAC-based phosphoproteomic approach to compare mammary cell lines with different Syk expression or catalytic activity [32] we identified potential Syk substrate proteins involved in cell-cell adhesion (E-Cdh, α-Ctn) and epithelial polarity (occludin, Scrib, Dlg, ZO3, claudin3, InaDL, MAGUK5, and Lin7C). These gatekeepers against cancer are hallmarks of tumor suppression [33]. Several observations indicated a role for Syk in intercellular contact formation [32,34]. We found that Syk colocalizes with E-Cdh at cell-cell contacts and that its activity is required for the proper localization of p120-Ctn at AJ [32]. Here, we investigated whether the E-Cdh/Ctn complex is directly phosphorylated and regulated by Syk and studied its consequences on the E-Cdh complex stability, intercellular adhesion, epithelial polarity, and cell migration and invasion using both cell lines and a conditional *Syk* knockout model in the mouse mammary gland.

## 2. Results

### 2.1. Syk Phosphorylates the E-Cadherin/Catenin Complex on Different Tyrosine Residues

**Figure 1:**
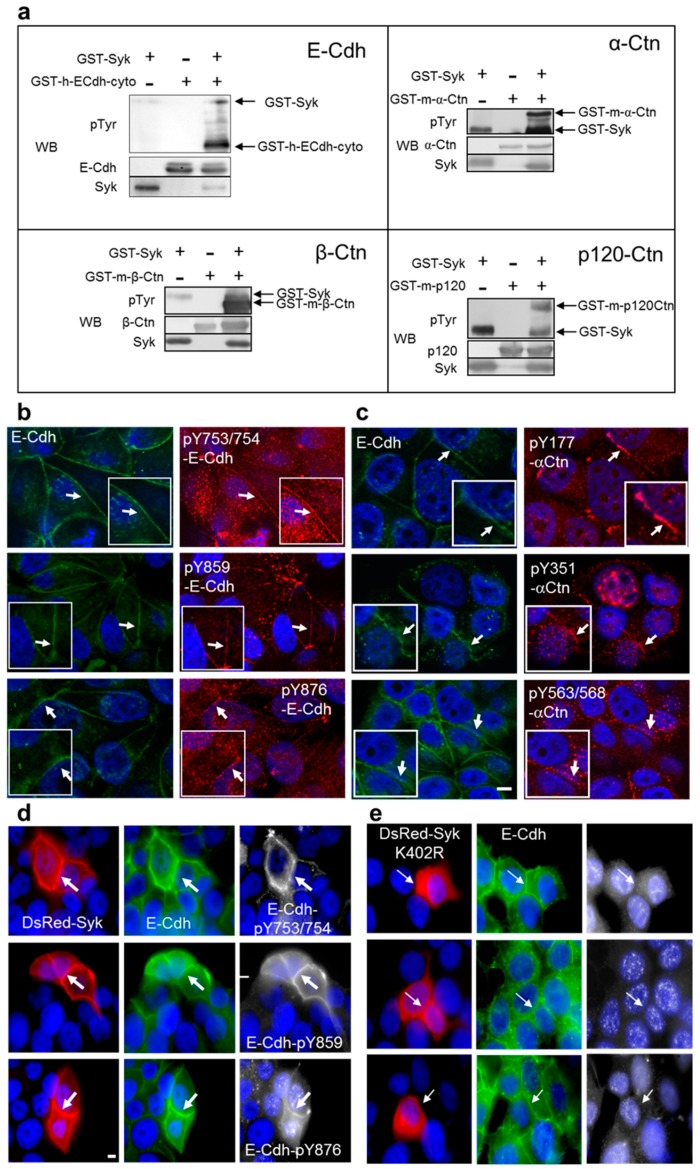
Spleen tyrosine kinase (Syk) phosphorylates E-cadherin and α-, β-, and p120-catenins and their phosphorylated forms localize at adherens junctions. (**a**) In vitro kinase reactions using nonradioactive ATP and recombinant GST-Syk, GST-E-Cdh-cyto (cytoplasmic domain), GST-α-Ctn, GST-β-Ctn, and GST-p120-Ctn, as indicated. Proteins were separated by SDS-PAGE and analyzed by Western blotting (WB). H, human; m, murine. (**b**,**c**) Immunofluorescence analysis of MCF7 cells using anti-E-Cdh (FITC/green) and antibodies against phosphorylated E-Cdh (Y753/754, Y859, or Y876) (b) or phosphorylated α-Ctn (Y177, Y351, or Y563/568) (c) (TRITC/red). Thick arrows indicate colocalization of endogenous E-Cdh with phosphorylated E-Cdh/α-Ctn at adherens junctions. Enlarged regions of interest are shown within the insets. (**d**,**e**) Immunofluorescence analysis of MCF7 cells transiently transfected with a plasmid encoding wild type (WT) (d) or kinase-dead (K402R) mutant (e) DsRed-Syk. Tyrosine phosphorylation of E-Cdh (d) and α-Ctn (e) at adherens junctions was assessed with the indicated antibodies (Cy5/white). Thin arrows indicate E-Cdh-positive cell junctions (FITC/green) in kinase-dead (K402R) DsRed-Syk-positive cells, but without E-Cdh and α-Ctn phosphorylation at the indicated tyrosine residues. DNA was visualized with Hoechst (blue). Scale bar: 10 μm.

Using quantitative phosphoproteomics and in vitro kinase assays with recombinant proteins, we previously reported that E-Cdh and α-Ctn are direct substrates of the Syk kinase [32]. Here, we performed in vitro kinase assays with the β-Ctn and p120-Ctn E-Cdh/Ctn complex components and demonstrated that E-Cdh, α-Ctn, β-Ctn, and p120-Ctn were all phosphorylated by Syk (Figure 1a), in addition to Syk autophosphorylation. These assays were performed in the presence of nonradioactive ATP allowing to analyze and identify the purified phosphorylated E-Cdh and Ctn peptides by mass spectrometry (Supplementary Figure S1a). Syk-mediated phosphorylation revealed the following tyrosine residues within E-Cdh (Y753/754, Y859, Y876), α-Ctn (Y177, Y351, Y563/568), and β-Ctn (Y30). Phosphorylations on E-Cdh Y876, α-Ctn Y177, α-Ctn Y563, and β-Ctn Y30 have been reported in high-throughput studies but without known effects (http://www.phosphosite.org/). Phosphorylation of E-Cdh at Y753/754 has been reported [35,36] and its consequences will be discussed below. We also identified the Syk-mediated phosphorylation of β-Ctn at Y142 (data not shown), a residue known to be phosphorylated by the Fer and Fyn kinases that is involved in regulating its interaction with α-Ctn [37]. β-Ctn phosphorylation at Y142 has recently been observed at centrosomes where it may regulate centrosomal cohesion [38]. In p120-Ctn, 16 residues were phosphorylated by Syk (data not shown), in agreement with its recognition as a highly phosphorylated protein [39].

We generated double affinity-purified antibodies that recognize specifically the phosphorylated forms of the peptides (Supplementary Figure S1b). In confluent MCF7 breast cancer cells (Syk+/E-Cdh+), baseline phosphorylation of E-Cdh (Figure 1b) and α-Ctn (Figure 1c) at these residues localized at AJ (arrows), but exhibited a low signal level. As reported for pY654-β-Ctn [40], a clearly discernible tyrosine-phosphorylation at AJ generally necessitates a stimulation of cells (e.g., with growth factors).

### 2.2. Exogenous Syk Expression and Oxidative Stress Increase E-Cadherin and Catenin Phosphorylation at Adherens Junctions

**Figure 2:**
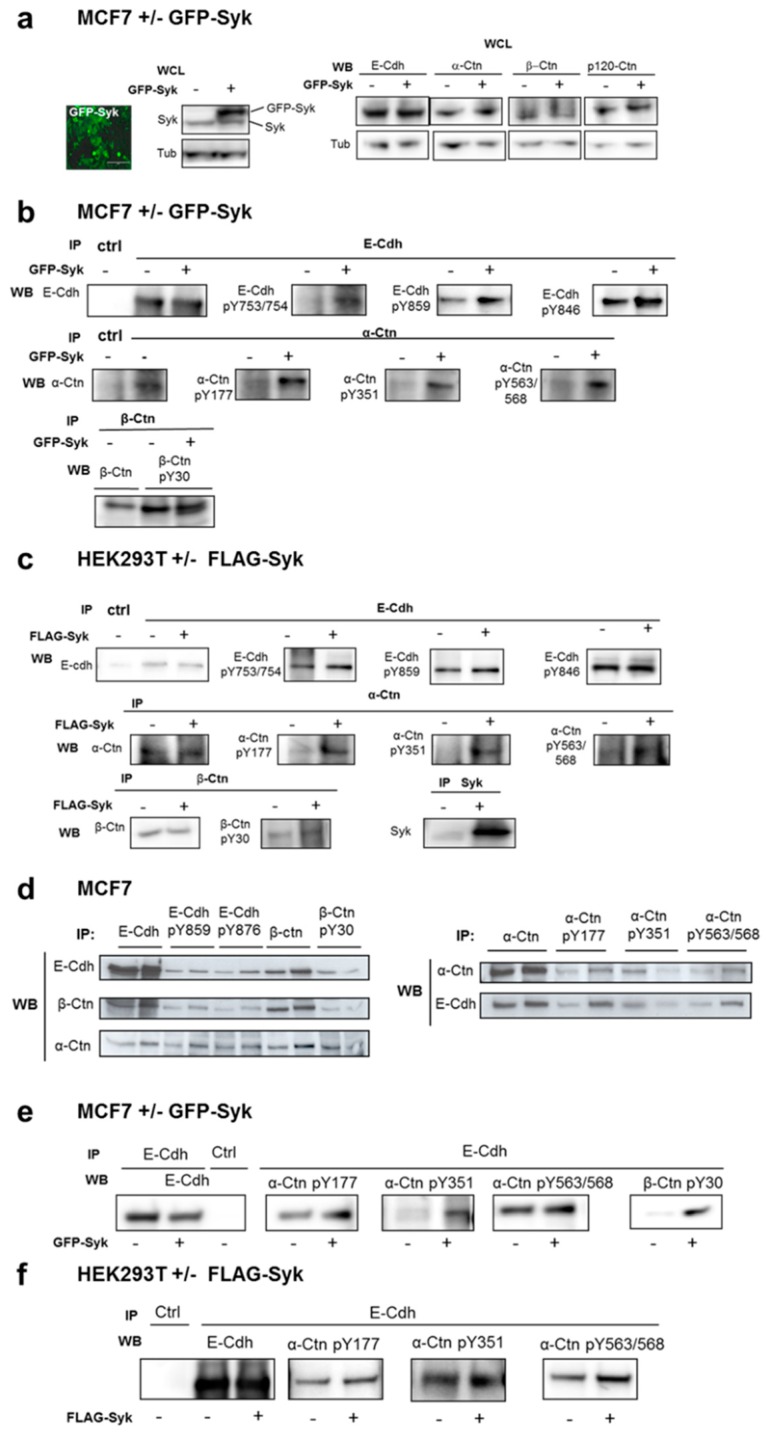
Exogenous Syk expression increases E-cadherin and catenin phosphorylation and interaction. (**a**) Representative image of MCF7 cells that stably express GFP-Syk. Scale bar: 200 μm. Whole cell lysates (WCL) of parental and GFP-Syk MCF7 cells were analyzed by Western blotting (WB) with anti-Syk, -E-Cdh, -α-, -β-, and -p120-Ctn antibodies. β-tubulin was used as loading control. (**b**) Protein lysates of nontransfected and GFP-Syk-expressing MCF7 cells were immunoprecipitated (IP) with anti-E-Cdh, -α- and -β-Ctn antibodies and analyzed by Western blotting to detect E-Cdh/Ctn phosphorylation at the indicated tyrosine residues. (**c**) Protein lysates of HEK293T cells transiently transfected or not with a FLAG-Syk plasmid were immunoprecipitated (IP) using anti-E-Cdh, -α- and -β-Ctn or -Syk antibodies and analyzed by Western blotting (WB). (**d**–**f**) E-cadherin and catenin interaction was evaluated by co-immunoprecipitation (IP) followed by Western blotting (WB) using the indicated antibodies against E-Cdh, α-, and β-Ctn and the different phosphorylated forms and protein lysates from parental MCF7 cells (d), nontransfected and GFP-Syk-expressing MCF7 cells (e), and HEK293T cells transiently transfected or not with a FLAG-Syk plasmid (f).

Syk can be activated (autophosphorylated) by its exogenous expression at higher levels and also by reactive oxygen species (ROS) because it plays a crucial role in oxidative stress signaling [41]. In agreement, exogenously expressed DsRed-Syk strongly localized at AJ and phosphorylation of E-Cdh (Figure 1d), α-Ctn, and β-Ctn (Supplementary Figure S2) was increased at AJ. Pixel intensity quantification indicated a 2-fold E-Cdh increase at AJ (Supplementary Figure S3). This could be the result of its increased accumulation/recruitment at cell-cell junctions, because total E-Cdh expression was not increased in FLAG-Syk and GFP-Syk-expressing cells (Figure 2a,b). Kinase-dead DsRed-Syk (K402R) expression in MCF7 cells did not induce detectable E-Cdh, α-Ctn, and β-Ctn phosphorylation at cell-cell junctions (Figure 1d,e). This suggests that Syk kinase activity is required for the phosphorylation of the E-Cdh/Ctn complex and for the accumulation of Syk at the cell membrane. Only β-Ctn-pY30 nuclear expression was not affected in DsRed-Syk K402R-expressing cells. The nuclear speckles of phosphorylated E-Cdh could correspond to E-Cdh cleaved cytoplasmic domain [42]. It has been reported that loss of membrane localization and aberrant nuclear E-Cdh expression correlates with invasion [43,44].

Similarly, in MCF7 cells treated with hydrogen peroxide (H_2_O_2_) or sodium peroxyvanadate (PV) to activate endogenous Syk, global phospho-tyrosine levels, and E-Cdh phosphorylated at Y753/754 and Y876 were strongly increased at AJ (Supplementary Figure S4). H_2_O_2_ and PV treatments did, however, not modify E-Cdh levels at AJ.

We confirmed these immunofluorescence results by Western blot and immunoprecipitation experiments in MCF7 cells stably expressing GFP-Syk (Figure 2a, left panel). Exogenous GFP-Syk expression did not affect the total E-Cdh and catenin levels (Figure 2a). This supports the idea that the increased E-Cdh levels at AJ in DsRed-Syk MCF7 cells are due to E-Cdh localization/recruitment at the plasma membrane (Supplementary Figure S3). Phosphorylation levels of E-Cdh, α-Ctn, and β-Ctn were significantly increased in GFP-Syk MCF7 cells (Figure 2b). We obtained similar results in HEK293T cells after transient transfection of FLAG-Syk (Figure 2c).

### 2.3. Syk-Mediated Phosphorylation of E-Cadherin Promotes its Interaction with Catenins and Regulates its Internalization in Early Endosomes

We then asked whether Syk-mediated phosphorylation stabilized or reduced the interactions between the E-Cdh/Ctn complex components. Phosphorylated E-Cdh, α-Ctn, and β-Ctn levels in nontransfected MCF7 cells represented only a minor part of the total proteins (Figure 2d). This fraction could represent the intercellular-localized subpopulation. Moreover, α-Ctn and β-Ctn were co-immunoprecipitated with total/phosphorylated E-Cdh; E-Cdh and α-Ctn with pY30-β-Ctn; and E-Cdh with phosphorylated α-Ctn (pY177, pY351, and pY563/568) (Figure 2d). This suggests that Syk-mediated phosphorylation of these proteins does not negatively affect the E-Cdh/catenin complex. In agreement, exogenous GFP- or FLAG-tagged Syk expression, respectively, in MCF7 (Figure 2e) and HEK293T (Figure 2f) cells increased catenin association with E-Cdh, indicating that these phosphorylations stabilize or promote their interaction.

**Figure 3:**
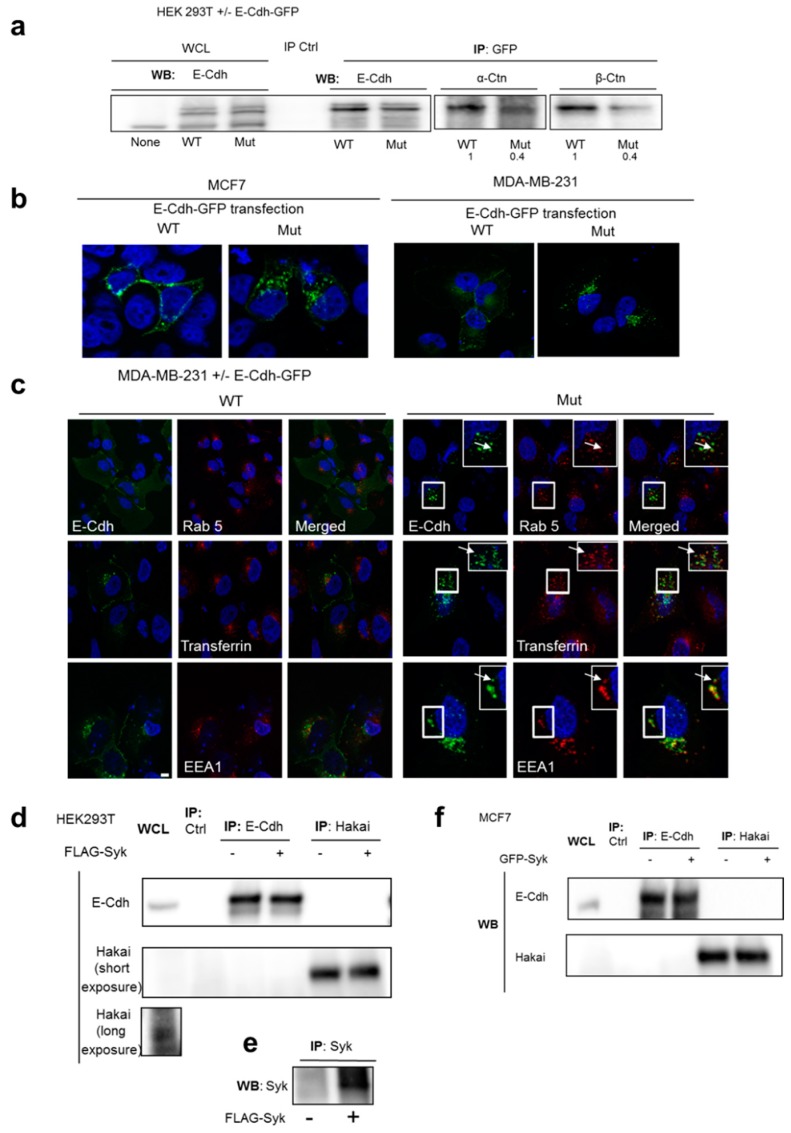
The E-cadherin mutant that cannot be phosphorylated by Syk displays reduced interaction with catenins and increased endocytosis, but not ubiquitination and degradation. (**a**) Protein lysates of HEK293T cells transiently transfected with plasmid encoding wild type (WT) or an E-Cdh-GFP mutant that cannot be phosphorylated by Syk (Y753, 754, 859, and 876 mutated into F) were immunoprecipitated (IP) with anti-GFP antibody and analyzed by Western blotting (WB) using anti-E-Cdh, -α-, and -β-Ctn antibodies. The immunoprecipitated proteins were quantified by densitometry relative to the WT band set to 1. (**b**) Localization of E-Cdh-GFP was assessed by immunofluorescence analysis of MCF7 and MDA-MB-231 cells transiently transfected with WT or mutant E-Cdh-GFP (4Y/4F). DNA was visualized with Hoechst (blue). (**c**) Characterization of the E-Cdh-GFP-positive vesicles in MDA-MB-231 cells using anti-RAB5 and -EEA1 (TRITC/red) antibodies and by incubation with fluorescent transferrin-Cy3 (red). Insets show enlarged cytoplasmic regions of cells that express mutant E-Cdh-GFP. Arrows indicate the colocalization of mutant E-Cdh-GFP with RAB5, EEA1, and transferrin (three markers of early endosomes). Scale bar: 10 μm. (**d**) Western blot (WB) analysis of Syk, E-Cdh and Hakai expression in immunoprecipitates (IP) from HEK293T cells transiently transfected or not with a FLAG-Syk plasmid (**e**) and in parental and GFP-Syk MCF7 cells (**f**). WCL, whole cell lysate.

Membrane-associated E-Cdh in mature junctions is quickly renewed by endocytosis, thereby tightly controlling the number of functional processes [12]. To investigate the role of Syk-mediated phosphorylation in E-Cdh dynamics, we transfected HEK293T cells with wild type (WT) or mutant E-Cdh-GFP in which the four identified tyrosine residues (Y753/754, Y859, Y876) could not be phosphorylated (Y/F point mutation, Mut). With comparable expression levels of WT and Mut E-Cdh-GFP (slightly above endogenous E-Cdh) (Figure 3a, left panel), co-immunoprecipitation of α-Ctn and β-Ctn with E-Cdh were reduced by ≈60% in cells expressing Mut E-Cdh-GFP. Immunofluorescence analysis of MCF7 (Syk^+^/E-Cdh^+^) and MDA-MB-231 (Syk^−^/E-Cdh^−^) breast cancer cells transfected with WT or Mut E-Cdh-GFP showed that WT E-Cdh was mainly localized at AJ, whereas Mut E-Cdh-GFP was predominantly expressed in intracellular vesicles (Figure 3b). These vesicles were positive for RAB5, a key regulator of vesicular trafficking during early endocytosis, fluorescent transferrin, and early endosome antigen 1 (EEA1) (Figure 3c), indicating that they were early endosomes. These data suggest that inhibiting the Syk-mediated phosphorylation of E-Cdh stimulates its internalization and turnover.

The Y753/754 residues are involved in E-Cdh interaction with the Hakai E3 ubiquitin-ligase, inducing ubiquitination of the E-Cdh complex [35,36]. This interaction depends on the direct recognition of the pY754 residue by Hakai [45]. In our experiments, Syk-mediated phosphorylation of these residues did not affect the overall E-Cdh levels (Figure 2b) and we did not observe any co-immunoprecipitation between E-Cdh and Hakai in exogenous Syk-expressing HEK293T and MCF7 cells (Figure 3d,f). This suggests that Syk is not involved in Hakai-mediated ubiquitination and degradation of E-Cdh after endocytosis.

### 2.4. Syk Positively Affects 2D and 3D Cell Aggregation

**Figure 4:**
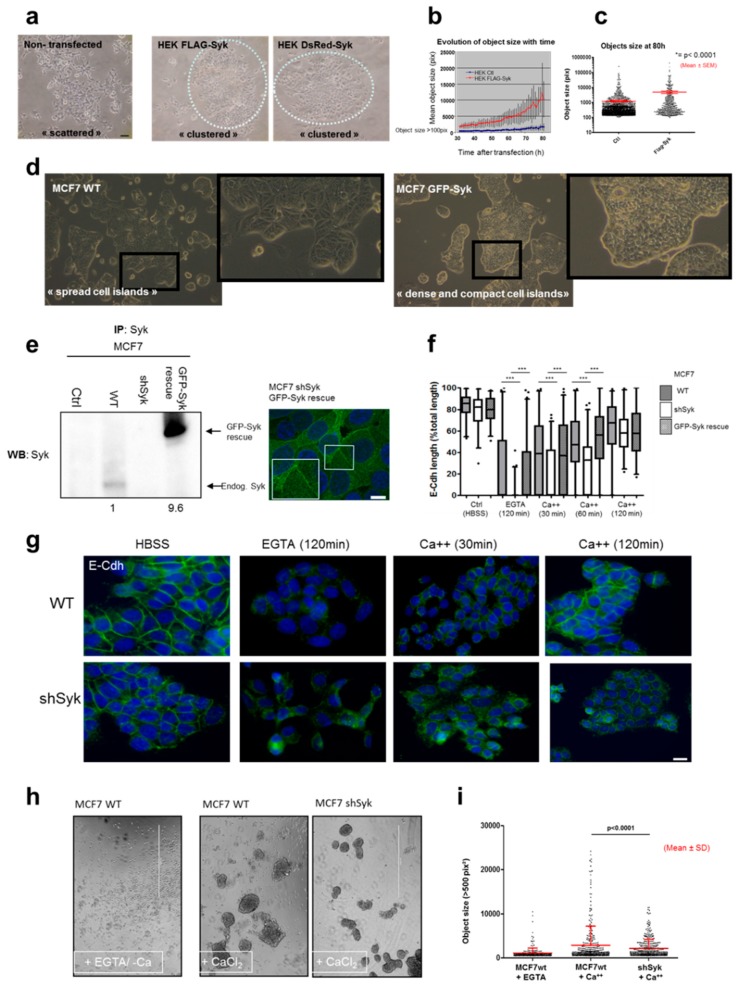
Syk promotes cell-cell adhesion and cell re-aggregation. Cell-cell adhesion was monitored by video-microscopy in HEK293T cells transiently transfected or not with a FLAG-Syk or DsRed-Syk plasmid (**a**–**c**). The mean object size (for cells with a size > 100 pixels) of transfected and control (Ctl) HEK293T cells was quantified over time and at the end point (80 h post-transfection). (a) Bright-field microscopy images of nontransfected and FLAG-Syk or DsRed-Syk transfected HEK293T cells. (b) Change of the mean object size over time after transfection. (c) Scatter plot showing the object size (mean ± SEM) at the end point; *** *p* value < 0.0001 (Student’s *t*-test). (**d**) Bright-field microphotographies of parental and GFP-Syk MCF7 cells seeded on cell culture plastic plates. (**e**) Lysates from parental (WT), MCF7 cells in which SYK was silenced (shSyk), and in shSyk MCF7 cells in which Syk expression was rescued by transfection of a GFP-Syk plasmid (GFP-Syk rescue) were immunoprecipitated (IP) and analyzed by Western blotting (WB) with anti-Syk antibodies. Compared with parental MCF7 cells, Syk was overexpressed (left panel; densitometric quantification) and localized at intercellular junctions (right panel) in GFP-Syk rescue cells. The inset shows an enlarged image of the plasma membrane regions where GFP-Syk is localized at cell-cell junctions. DNA was detected with Hoechst (blue). Scale bar: 10 μm. (**f**,**g**) Cell-cell junction dynamic monitoring in parental (WT), shSyk, and GFP-Syk rescue MCF7 cells after incubation with EGTA in HBSS medium for 2 h (junction disruption), followed by switch to DMEM medium containing CaCl_2_ (for 30, 60, and 120 min) to allow adherens junction restoration. Cells seeded on coverslips were processed for immunofluorescence with an anti-FITC-E-Cdh antibody to measure the length of E-Cdh-positive cell membrane relative to the total cell border length at the indicated time points (f); *** *p* value < 0.001 (Student’s *t*-test). Representative images of parental (WT) and shSyk MCF7 cells at the indicated time points (g). DNA was visualized with Hoechst (blue). Scale bar, 10 μm. (**h**) Parental (WT) and shSyk MCF7 cells were trypsinized and allowed to re-aggregate for 18 h on a cell shaker (80 rpm at 37 °C) in E-Cdh stabilizing (with Ca^++^) or destabilizing (with EGTA, without Ca^++^) conditions. (h) Bright field images of cell aggregates and isolated cells at the end point. (**i**) The number and size (>500 pix^2^) of the cell objects were determined using the Cellomics-based bioimaging analysis. Scatter plot showing the mean ± SD of the object size value at the end point; *** *p* value < 0.0001 (Students *t*-test).

To determine the role of Syk in cell-cell adhesion, we transfected HEK293T cells with FLAG-Syk or DsRed-Syk and monitored them by live video-microscopy. Syk-transfected cells exhibited a clustered morphology with most cells touching each other, whereas nontransfected cells showed a scattered morphology (Figure 4a). Video-microscopy analysis confirmed the cell-cell seeking and gathering. Object size quantification (>100 pixels) showed a progressive increase of the mean object size in transfected cells over time (Figure 4b) and at the 80 h endpoint (Figure 4c). Similarly, MCF7 cells grew as well-spread islands, whereas GFP-Syk MCF7 cells appeared as dense and compact cell masses (Figure 4d).

We then monitored the AJ dynamics by counting the length of the E-Cdh signal relative to the total cell border length in MCF7, shSyk MCF7, and shSyk MCF7 cells expressing a rescue GFP-Syk protein (shSyk-GFP-Syk MCF7) (Figure 4e), after incubation with the Ca^++^ chelator EGTA (to destabilize E-Cdh homophilic interaction) and after Ca^++^ addition. The cell-membrane-associated E-Cdh signal decrease after incubation with EGTA was significantly faster and its reappearance was significantly slower after Ca^++^ addition in shSyk MCF7 cells than in WT and shSyk-GFP-Syk MCF7 rescue cells (Figure 4f,g). Opposite results were obtained with GFP-Syk MCF7 cells (Supplementary Figure S5).

As cell-cell adhesion is mainly a three-dimensional phenomenon, we also performed 3D re-aggregation assays in which trypsinized parental and shSyk MCF7 cells were allowed to re-aggregate in E-Cdh destabilizing (EGTA) conditions and after Ca++ addition. Microscopy images revealed the presence of fewer but bigger cell aggregates in parental MCF7 cells compared with shSyk MCF7 cells after Ca++ addition (Figure 4h). Quantification of the number and size (>500 pix^2^) of the cell objects confirmed this difference (Figure 4i), indicating that Syk promotes E-Cdh recruitment and stabilization at AJ.

### 2.5. Syk Negatively Affects Cell Migration, Invasion, and Clonogenicity, and Maintains Epithelial Integrity

**Figure 5:**
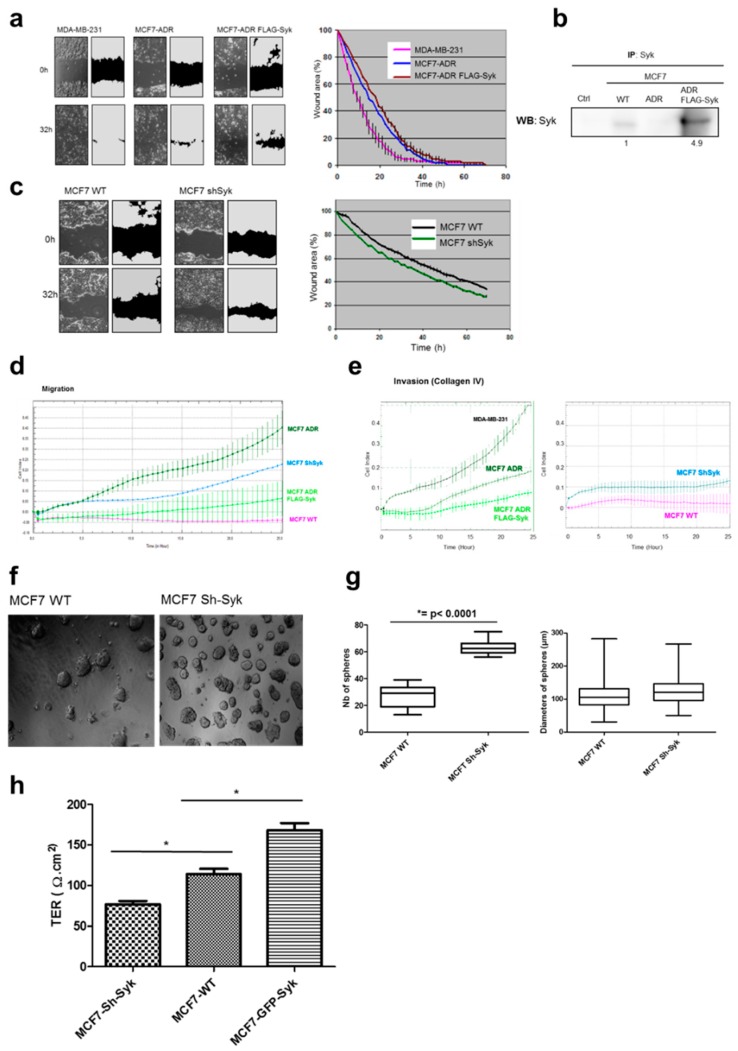
Enhanced Syk expression hinders cell migration and invasion, reduces the clonogenic potential, and maintains the epithelial integrity. (**a**) Confluent cell layers were scraped and cell migration into the wound was monitored by time-lapse microscopy for 70 h to quantify cell migration over time (right panel). MDA-MB-231 cells were used as a positive control of migrating, invasive breast cancer cells. (**b**) Parental (WT) MCF7, MCF7/ADR (Syk-/E-Cdh-), and FLAG-Syk-expressing MCF7/ADR cell lysates were immunoprecipitated (IP) and analyzed by Western blotting (WB) with anti-Syk antibodies. The numbers under the lanes indicate the relative expression levels determined by densitometry. (**c**) The same experiment as in (a) was performed with parental (WT) and shSyk MCF7 cells. Error bars represent the SD of triplicate wells. (**d**,**e**) Cell migration and invasion in collagen IV were assessed using the xCELLigence real-time cell analysis technology (Roche). Comparison of cell migration (d) and cell invasion (e) in MCF7/ADR, FLAG-Syk MCF7/ADR, parental MCF7 (WT), and shSyk MCF7 cells using the Cell Index (CI) as unit. Error bars represent the SD of triplicate wells. (**f**,**g**) Parental (WT) and shSyk MCF7 cells were seeded in a 3D Matrigel layer. At day 14 post-seeding, the resulting spheres were observed by bright-field microscopy (f) and their number and diameter were quantified (g) *** *p* value < 0.0001 (Mann–Whitney test). (**h**) Transepithelial resistance (TER) measurement of highly confluent and tight monolayers of parental (WT), shSyk, and GFP-Syk MCF7 cells. Results represent the mean ± SD of triplicate wells; * *p* value < 0.05 (paired *t*-test).

E-Cdh activity deregulation is associated with increased cell migration and the acquisition of an invasive phenotype. We therefore tested the consequences of Syk transfection and knockdown in the 2D wound healing assay using Syk^−^ MCF7-ADR cells that express or not FLAG-Syk (Figure 5a,b) and in MDA-MB-231 (Syk^−^/E-Cdh^−^) motile/invasive cells. Cell migration was followed up to 70 h and quantified. FLAG-Syk expression slowed down wound healing in MCF7-ADR cells (Figure 5a). Conversely, the wound healing capacity was significantly increased in shSyk MCF7 compared with parental MCF7 cells (Figure 5c).

As in this assay cells may migrate collectively, retaining cell-cell contacts, we also performed Boyden chamber migration and invasion assays. Continuous quantification for 25 h of cell migration showed that FLAG-Syk-expressing MCF7-ADR cells migrated significantly slower than the parental cells (Figure 5d). Conversely, *SYK* knockdown significantly increased MCF7 cell migration. Similar results were obtained for the invasive capacity through collagen type IV (Figure 5e).

Analysis of the *SYK* knockdown effect on cell proliferation and outgrowth in anchorage-dependent conditions (Matrigel) showed that parental and shSyk MCF7 cells formed spherical clones with comparable morphology and diameter, but the number of clones was significantly higher in shSyk cells (Figure 5f,g), indicating that Syk negatively affects the clonogenic potential.

Finally, we confirmed the role of Syk in promoting E-Cdh-mediated epithelial integrity by measuring the transepithelial resistance (TER) of highly confluent and tight monolayers of parental, shSyk, and GFP-Syk MCF7 cells. TER was reduced in shSyk and increased in GFP-Syk cells (Figure 5h).

### 2.6. Enhanced Syk Expression Increases the Interaction between the E-Cadherin/Catenin Complex with Zonula Occludens Proteins and the Actin Cytoskeleton

**Figure 6:**
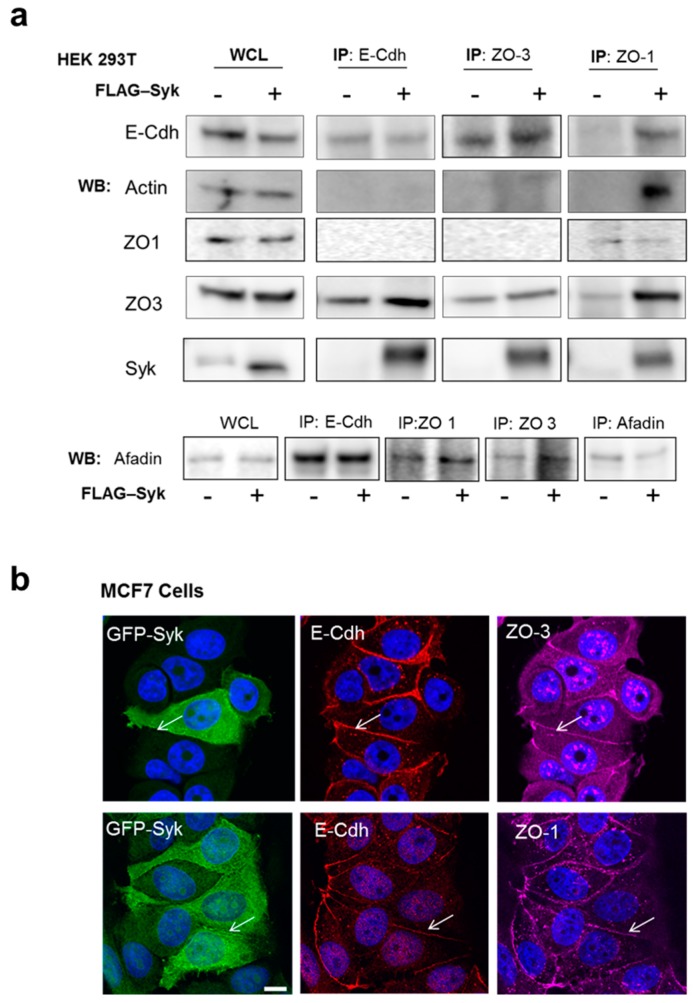
Enhanced Syk expression promotes the interaction between the E-cadherin/catenin complex, zonula occludens proteins, and the actin cytoskeleton. (**a**) Whole cell lysates (WCL) and immunoprecipitated proteins (IP) from HEK293T cells transiently transfected or not with a FLAG-Syk plasmid were analyzed by Western blotting (WB) with the indicated antibodies. (**b**) Immunofluorescence analysis of GFP-Syk MCF7 cells with anti-E-Cdh (TRITC/red), -ZO-1 (upper) and -ZO-3 antibodies (lower) (Cy5/purple). White arrows indicate GFP-Syk colocalization with E-Cdh and ZO-1 or ZO-3 at cell-cell junctions. DNA was stained with Hoechst (blue). Bar, 10 μm.

To understand how Syk stabilizes cell-cell interactions and epithelial integrity, we performed co-immunoprecipitation experiments using parental and FLAG-Syk HEK293T cells and antibodies against junctional complex components involved in the interaction of E-Cdh/Ctn complexes with the actin cytoskeleton (Figure 6a). Syk transfection promoted the interaction of E-Cdh with zonula occludens 3 (ZO3) and Syk. It also promoted E-Cdh, actin, ZO3, and Syk co-immunoprecipitation with ZO1 and thus appears to be a major linker between the E-Cdh/Ctn complexes and the actin cytoskeleton. ZO1 and ZO3 also co-immunoprecipitated afadin, an actin filament-binding protein that colocalizes at cadherin-based AJ. Immunofluorescence analysis of GFP-Syk MCF7 cells showed that ZO1 and ZO3 partly colocalized with E-Cdh and Syk (Figure 6b). This suggests that they might locally interact and form a complex that links E-Cdh/Ctn with the actin cytoskeleton.

### 2.7. *Syk* Loss Affects Alveolar Epithelial Cell Differentiation in Lactating Mammary Glands and Disrupts E-Cadherin Localization at Intercellular Junctions

**Figure 7:**
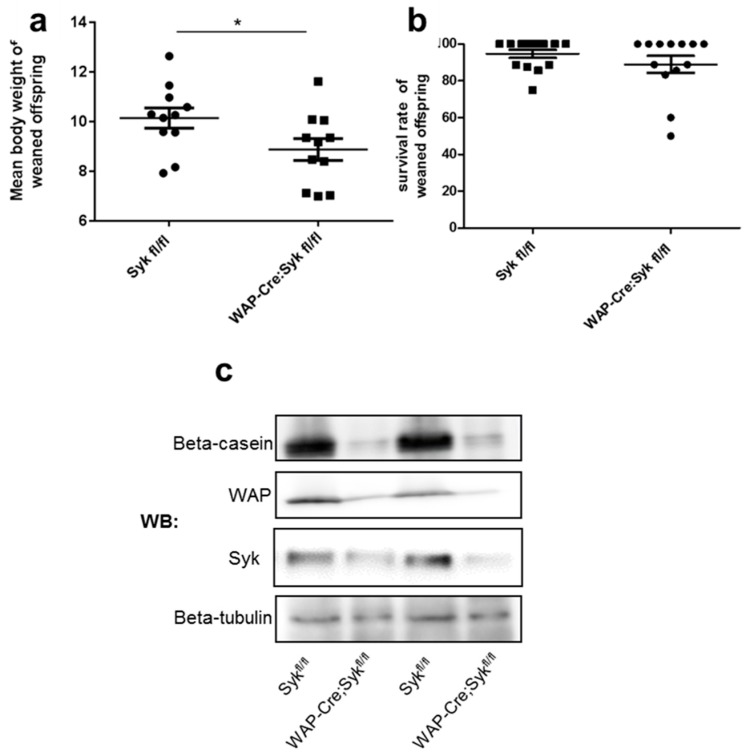
Reduced weight of the weaned offspring of mice with conditionally ablated Syk in the mammary glands is associated with decreased milk protein expression. (**a**) Body weight in grams (mean ± SEM) of the weaned offspring of *Syk*^fl/fl^ (control) and Wap-Cre;*Syk*^fl/fl^ mice (*n* = 11 mice/group; * *p* < 0.05, Mann–Whitney test). (**b**) Survival rate (mean ± SEM) of *Syk*^fl/fl^ and Wap-Cre;*Syk*^fl/fl^ offspring at weaning (*n* = 14 for *Syk*^fl/fl^ mice and *n* = 13 for Wap-Cre;*Syk*^fl/fl^ mice). (**c**) Representative Western blot image of *Syk*^fl/fl^ and Wap-Cre;*Syk*^fl/fl^ mammary epithelial cell lysates from four independent mothers at weaning after two pregnancies probed with anti-β-casein, anti-WAP antibodies. Syk expression levels were verified by Western blot (c) and by immunohistochemistry (Supplementary Figure S6d,e). (β-tubulin: loading control).

**Figure 8:**
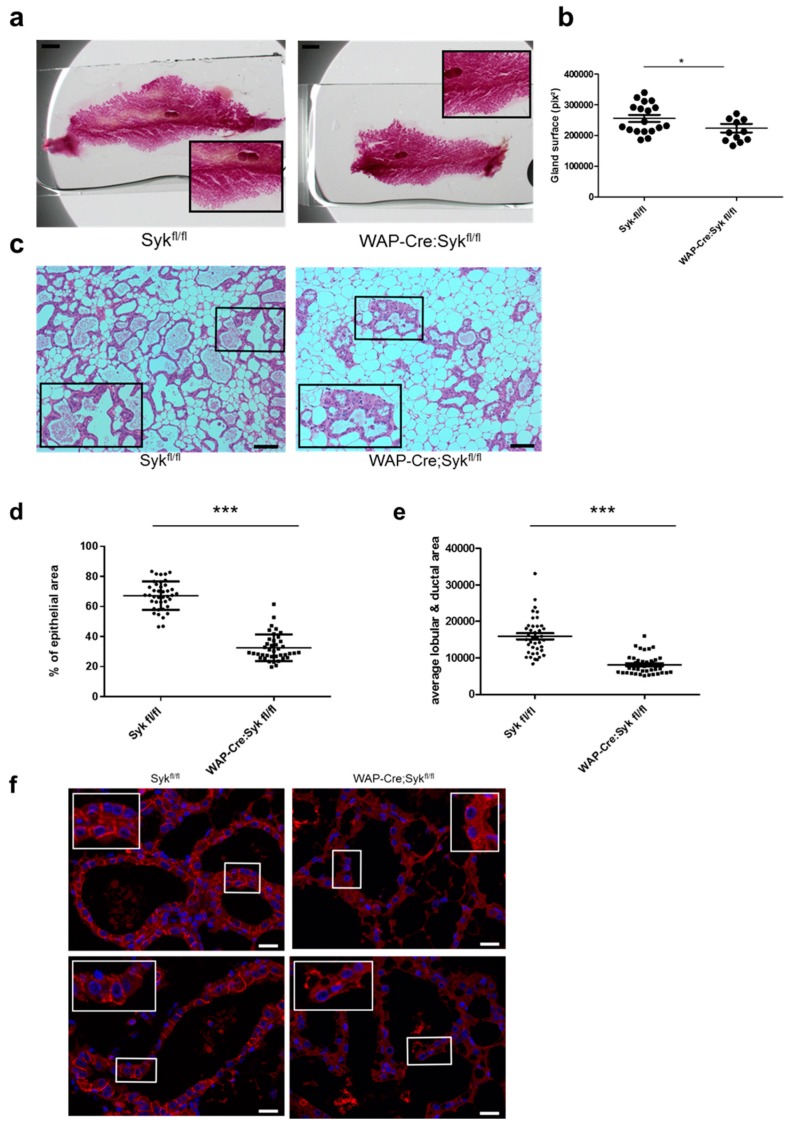
Syk expression loss negatively affects epithelial morphogenesis and E-cadherin localization at cell-cell junctions in lactating mammary glands. (**a**) Representative carmine red staining performed of *Syk*^fl/fl^ and Wap-Cre;*Syk*^fl/fl^ inguinal mammary glands at weaning after two pregnancies (3.8× magnification). Insets show an enlarged part of the mammary gland enclosing a lymph node (15× magnification). Scale bar: 100 pix. (**b**) The total inguinal mammary gland area was quantified with the ImageJ software (*n* = 18 for *Syk*^fl/fl^ and *n* = 11 for Wap-Cre;*Syk*^fl/fl^ mice; mean ± SEM; * *p* < 0.05, Mann–Whitney test) in pix^2^. (**c**) Hematoxylin and eosin staining of inguinal glands from *Syk*^fl/fl^ or Wap-Cre; *Syk*^fl/fl^ mice at weaning. Scale bar: 10 μm. (**d**) Percentage of mammary epithelium area (ducts and lobules) relative to the total mammary gland area in each studied field (epithelium and stroma). (**e**) Mean mammary epithelium area (ducts and lobules) in each studied field in pix^2^ (*n* = 8 for *Syk*^fl/fl^ and Wap-Cre;*Syk*^fl/fl^, five fields measured for each mouse; mean ± SEM; *** *p* < 0.001, Student’s *t*-test). (**f**) *Syk*^fl/fl^ (control) and Wap-Cre;*Syk*^fl/fl^ mammary gland tissue sections at weaning (after two pregnancies) were immunostained to assess E-Cdh expression (TRITC/red). DNA was stained with Hoechst (blue). Scale bars: 10 μm.

E-Cdh is essential for the development of the epithelial architecture and phenotype by promoting tight connections between epithelial cells [46]. To verify the role of Syk in E-Cdh/Ctn complex stabilization in vivo, we examined the consequences of *Syk* loss during mouse mammary gland development. As *Syk* deletion is perinatal lethal in mice [47,48], we deleted *Syk* exclusively in the differentiating mammary gland by breeding floxed *Syk* mice (Syk^fl/fl^) with Wap-Cre transgenic mice that express the Cre-recombinase gene under the control of the lactogenic hormone-induced whey acidic protein (*Wap*) gene promoter [49]. Genomic PCR of epithelial cells isolated from Wap-Cre;Syk^fl/fl^ *Syk* conditional knockout (cKO) lactating mammary glands at weaning after two pregnancies [49] confirmed excision of the *Syk* allele (Supplementary Figure S6a). The specificity of the anti-Syk antibody was verified on paraffin sections of Syk− (MDA-MB-231), Syk+ (MCF7), and Syk-transfected (MCF7-GFP-Syk) cell lines (Supplementary Figure S6b). Syk expression was strongly present in the mammary gland luminal epithelium and in lymphocyte subsets in the lymph nodes (Supplementary Figure S7). We confirmed the loss of Syk expression in the mammary epithelium of cKO *Syk* mice by immunohistochemical analysis (Supplementary Figure S6d,e). Macroscopically, *Syk* cKO pups appeared identical to *Syk*^fl/fl^ pups at weaning. However, their body weight was lower, probably due to the decreased expression of milk proteins (β-casein and WAP) by epithelial cells of the mother’s lactating mammary glands (Figure 7a–c). Moreover, the overall size of carmine red-stained whole-mount lactating mammary gland preparations at weaning after two pregnancies was significantly lower in *Syk* cKO mice compared with *Syk*^fl/fl^ control mice (Figure 8a,b). In virgin mice, the epithelium of the adult mammary gland embedded in the stroma is traditionally described as a bilayer of apically oriented luminal secretory cells that undergo functional differentiation in pregnancy to produce milk, and of basally contractile myoepithelial cells that participate in milk delivery [50]. Microscopy analysis of the morphology and organization of the mammary epithelium (ducts and lobules) after hematoxylin/eosin (H&E) staining of lactating mammary glands at weaning showed the presence of fewer epithelial areas (ducts and lobules) within the stroma and significantly less secretory units, composed of ducts and lobules, in *Syk* cKO than in *Syk*^fl/fl^ mice (Figure 8c–e). The alveoli were condensed, disorganized, and less developed with small lumina, suggesting perturbed alveologenesis of the lactating mammary gland. H&E staining of involuting mammary gland tissue sections (3 days after weaning) in *Syk*^fl/fl^ and *Syk* cKO Syk mice displayed the same perturbed phenotype as during lactation (Supplementary Figure S8). Analysis of E-Cdh expression in mammary gland showed that E-Cdh localization at the intercellular junctions of mammary epithelial cells was lost in *Syk* cKO mammary glands, whereas E-Cdh cytoplasmic expression was not affected (Figure 8f). These in vivo data support the in vitro observations indicating that Syk stabilizes the E-Cdh/Ctn complex at the intercellular junctions.

## 3. Discussion

In this study, we investigated the regulation of the E-Cdh/Ctn complex by Syk phosphorylation, its positive consequences on intercellular adhesion and epithelial integrity, and its negative effects on cancer cell migration and invasion.

It has been reported that the E-Cdh/Ctn complex is regulated by phosphorylation at tyrosine (e.g., by Src, EGFR, or FGFR) and serine/threonine residues (e.g., by CK1&2, GSK3, PKCα&ℇ) and dephosphorylation (e.g., by PP1α, PP2A, PTEN) that positively or negatively affect the formation and stability of intercellular junctions [3,18]. We found that Syk phosphorylates not only E-Cdh and α-Ctn, but also β-Ctn and p120-Ctn on tyrosine residues, the majority of which remain currently unknown and their function undefined (http://www.phosphosite.org/). Moreover, we observed that E-Cdh/Ctn tyrosine phosphorylation occurs predominantly at AJ and is increased upon enhanced exogenous Syk expression or activation by oxidative stress. Whereas oxidative stress may compromise intercellular junctions and reduce TER resistance [51,52], H_2_O_2_ and PV did not disassemble E-Cdh-positive AJ in our experimental conditions. This may be due to Syk presence and activity because tyrosine kinases are critical for junction reassembly after oxidative stress [51,53].

We found that tyrosine-phosphorylated E-Cdh/Ctn complexes exhibit an increased interaction upon enhanced Syk expression, corroborating the hypothesis that Syk-mediated phosphorylation stabilizes/enhances their interaction at AJ. Indeed, the non-phosphorylatable GFP-E-Cdh mutant showed a decreased interaction with endogenous α-Ctn and β-Ctn and was predominantly localized in RAB5/EEA1-positive early endosomes, whereas WT E-Cdh remained at AJ.

The E-Cdh Y753/754 residues are part of a triple YYY sequence involved in the interaction with Hakai, a c-Cbl-like protein that ubiquitinates and induces endocytosis of the E-Cdh complex [35]. Phosphorylation at Y754 by Src-family kinases seems to be necessary and sufficient for these processes [45]. In our experiments, enhanced Syk expression induced phosphorylation of both Y753 and 754, but we did not observe any interaction between Syk and Hakai, or a reduction of the E-Cdh levels.

In 2D-cell cultures, FLAG-Syk and GFP-Syk transfection induced clustering and compaction of scattered cells. Sung and collaborators [54] found that following *SYK* silencing, cells showed EMT-related phenotypic changes, such as cell morphology and size modification in Matrigel and collagen I gels and upregulation of vimentin expression. Pathway analysis indicated that the genes differentially expressed after *SYK* knockdown were related to EMT. During EMT, cell-cell adhesions are dissolved, and the actin cytoskeleton is reorganized. E-Cdh/Ctn [8] and Syk [55] are involved in the actin cytoskeleton reorganization. Our experiments indicate that Syk positively affects the interaction between the E-Cdh/Ctn complex with zonula occludens proteins and the actin cytoskeleton. Enhanced Syk expression promoted E-Cdh/Ctn complex interaction with actin-binding proteins (ZO-1&-3, AF6/afadin) that could form an extra bridge between E-Cdh and actin [3]. In such way, Syk stabilizes intercellular adhesions and epithelial polarity. Consequently, Syk loss in breast cancer might promote breast cancer cell migration and invasion, and malignant tumor progression [19,20,29].

We conditionally inactivated the *Syk* gene in the mammary gland (Wap-Cre;Syk^fl/fl^ mouse strain). We observed that Syk is strongly expressed in luminal but not myo-epithelial cells of control mouse mammary gland epithelium. *Syk* loss was associated with a significant decrease in offspring weight at weaning, probably due to a decreased milk production as suggested by the reduced expression of the β-casein and WAP milk proteins in luminal epithelial cells of lactating mother glands. The overall mammary gland size of *Syk* cKO lactating mice was smaller than in control mice at weaning. Microscopic examination demonstrated a significantly reduced epithelial area (relative to the stromal area) that appeared disorganized with condensed and less developed alveoli, resembling that of the involuted glands after weaning and thus suggesting a disorder in alveologenesis. Moreover, *Syk* loss correlated with the disruption of E-Cdh localization at AJ in mammary epithelial cells, confirming our in vitro observations that Syk stabilizes the E-Cdh/Ctn complex at intercellular junctions. Mice lacking Syk (*Syk*^−/−^) die perinatally [47,48] due to the failure to separate the emerging lymphatic vessels from blood vessels [56]. Interestingly, the loss of only one Syk allele increases epithelial cell proliferation and ductal branching and invasion through the mammary fat pad during puberty [54]. However, this study was based on *Syk*^+/−^ virgin mice in which the entire fat pad is filled with a regularly spaced set of primary and secondary ducts [57] and in which Syk expression was reduced (but not fully lost) not only in the mammary glands. Moreover, the pathways regulating ductal and alveolar morphogenesis are completely different [58]. Our study is based on the complete *Syk* ablation in the mammary gland from mid-gestation when alveolar buds should form, grow, and differentiate into milk-secreting alveoli at the end of pregnancy. During lactation, alveoli are fully mature and luminal cells synthesize and secrete milk components into the lumina [50].

Interestingly, the phenotype of Wap-Cre;Syk^fl/fl^ mice is similar to that of MMTV-Cre;E-Cdh^fl/fl^ mice [59]. After normal mammary gland development during pregnancy, mutant E-Cdh adult mothers cannot feed their offspring due to a drastically reduced production of milk proteins. E-Cdh absence affects the terminal differentiation program of the lactating mammary gland, suggesting an essential role for E-Cdh in the function of differentiated alveolar epithelial cells. Our data demonstrate for the first time that *Syk* loss negatively affects E-Cdh localization at AJ and mammary gland development and functionality. Consequently, the loss of either *Syk* or *E-Cdh* induces a similar phenotype in the lactating mammary gland.

This study not only reveals the role of Syk in the maintenance of the mammary gland integrity. Loss of epithelial organization leads to EMT and invasion in breast cancer. Reduced Syk expression in breast tumors is associated with distant metastasis and poor prognosis and Syk is therefore considered to be a potential tumor suppressor and antimetastasis gene in human breast cancer [23,24,60]. Syk expression is progressively lost from normal to hyperplasia and DCIS (Ductal Carcinoma In Situ) to invasive tissue [23,24] and attributed to its promoter hypermethylation [22] and allelic loss [61]. Syk expression and activity are not restricted to the mammary gland epithelium as it is also present in the airway luminal [62] and prostate epithelium [63]. Hypermethylation of the *SYK* promoter leads to *SYK* silencing in human lung carcinoma and both are independent biomarkers of lung cancer development and metastatic spread [64]. Syk is weakly expressed in non-small-cell lung cancer (NSCLC) and its expression level inversely correlates with patient survival [65]. Syk depletion in K-Ras-dependent NSCLC cells results in loss of E-Cdh expression, indicative of EMT [66].

In contrast to epithelial cells in which Syk behaves as a tumor suppressor, Syk exhibits a rather oncogenic function in hematopoietic cells [29]. Proteomic and genetic approaches allowed to recognize Syk as an acute myeloid leukemia (AML) target [67]. Syk is a critical regulator of FLT3, the most commonly mutated kinase in AML. Its overexpression promotes transformation and resistance to FLT3-targeted therapy [68]. Syk plays also an important function in mantle cell lymphoma as a B cell receptor-associated kinase that activates the Bruton tyrosine kinase, a target of the ibrutinib inhibitor [69]. Such a dual role has been observed also with other kinases [70], but the comprehension and eventual reconciliation of these apparent contradictory functions are crucial for its eventual validation as a biomarker or therapeutic target. Our observations contribute to the understanding of how Syk negatively affects breast cancer invasion via the E-Cdh-based intercellular adhesion that is not present in hematopoietic cells.

In conclusion, this study demonstrates that Syk is actively involved in the maintenance of the epithelial integrity of the mammary gland via the stabilization of the E-Cdh/Ctn complex through phosphorylation, thereby inhibiting cell migration and malignant tumor invasion.

## 4. Materials and Methods

### 4.1. Cell Culture

MCF7 and MDA-MB-231 human breast cancer cells and HEK-293T cells were obtained from the ATCC and authenticated by STR analysis (Eurofins/MWG, Ebersberg, Germany). The adriamycin-resistant and Syk-negative MCF7/ADR cell line (also called NCI/ADR-RES) was from the Lombardi Cancer Center (Georgetown University, Washington, DC, USA) and was stably transfected with the FLAG-Syk construct. The shSyk MCF7 cell line [32] was stably transfected with the pEGFP-Syk-rescue-mutant and selected (800 µg/mL geneticin). MCF7 cells were stably transfected with the pEGFP-Syk plasmid and sorted with a FACSAria flow cytometer. Cells were cultured in Dulbecco’s modified Eagle’s medium (DMEM) supplemented with 10% fetal bovine serum (called complete medium) and kept under a humidified atmosphere at 5% CO_2_. To generate oxidative stress, cells were incubated with H_2_O_2_ (10 mM) or pervanadate (PV) (1 mM NaVO_4_ + 1 mM H_2_O_2_) (Sigma, St. Quentin Fallavier, France) in complete medium at 37 °C for 15 min.

### 4.2. Plasmids and Transfection

The FLAG-tagged Syk [20], the pEGFP-Syk [71], the mutant K402R DsRed-Syk [72], and E-cadherin-GFP (Addgene, Watertown, MA, USA) [73] constructs were previously described. The pEGFP-Syk-rescue mutant construct was obtained by generating a silencing mutation (CATCAT**CAG**TCAGAA to CATCAT**TTC**TCAGAA) in the SYK sequence using the QuikChange Site-Directed Mutagenesis Kit (Agilent Technologies, Santa Clara, CA, USA) and confirmed by sequencing.

Mutagenesis of the four tyrosine residues (753/754, 859, 876) of human E-cadherin-GFP to phenylalanine (Y/F) was performed using the Quick Change Site-Directed Mutagenesis Kit (Agilent Technologies) and confirmed by sequencing. FuGene6 (Promega Madison, Fitchburg, WI, USA) or Superfect (Qiagen, Courtaboeuf, France) were used for transfections.

### 4.3. Antibodies and Reagents

The following antibodies were used: (FITC-coupled) mouse anti-E-cadherin (36E), mouse anti-alpha-catenin and mouse anti-beta-catenin (BD Biosciences, Le Pont de Claix, France); mouse anti-Syk (clone 4D10), rabbit anti-Syk (clone N19), mouse anti-β-casein (clone FL-231), and goat anti-WAP (Santa Cruz Biotechnology, Dallas, TX, USA); rabbit anti-ZO-1 (Invitrogen, Carlsbad, CA, USA); rabbit anti-ZO-3 (Genetex, Irvine, CA, USA); rabbit anti-afadin, rabbit anti-RAB5 (C8B1), and rabbit anti-EEA1 (C45B10) (Cell Signaling Technology, Danvers, MA, USA); rabbit anti-Hakai (Bethyl Laboratories, Montgomery, TX, USA); rabbit anti-GFP (Torrey Pines Biolabs, Secaucus, NJ, USA); mouse anti-β-tubulin and mouse anti-SMA (Sigma); mouse anti-cytokeratin-8 (1E8) (AbCam, Cambridge, UK); and mouse-anti-pTyr (4G10) (Upstate Biotech Inc.)

Cy3-labeled human transferrin was kindly provided by B. Beaumelle (IRIM, Montpellier, France) [74]. Polyclonal rabbit anti-pY-Cdh and anti-pY-Ctn antibodies were raised against phosphorylated peptides (described in Supplementary Figure S1B) and validated by ELISA. Antisera were affinity-purified against nonphosphorylated and phosphorylated peptides (Eurogentec, Sart-Tilman, Belgium).

### 4.4. In Vitro Kinase Assays and Mass Spectrometry

Recombinant GST-h-E-Cdh-Cyto (cytosolic domain), GST-m-α-Ctn, GST-m-β-Ctn, and GST-m-p120 (provided by M. Duñach/A. Garcia De Herreros, Barcelona, Spain) were produced in *Escherichia coli* (BL21) as glutathione-S-transferase (GST) fusion proteins, and purified by affinity chromatography on glutathione–Sepharose columns (Sigma) [32]. Kinase reactions were performed by incubating 2.5 µg of GST-substrate protein with 100 ng recombinant GST-Syk (BPS Bioscience, San Diego, CA, USA) in 50 µL reaction buffer in the presence of cold (nonradioactive) ATP for 2 h [32]. Proteins from the kinase reactions were precipitated with acid buffer [1:10 2.5% SDS, 1:10 gelatin (Sigma), 8:10 30% TCA] at 4 °C for 1 h, washed in 15% TCA and boiled in Laemmli buffer with Tris, separated by SDS-PAGE, transferred to PVDF membranes and analyzed by Western blotting and detection with ECL (Amersham Biosciences, Little Chalfont, UK). Protein bands from the nonradioactive kinase reactions were in-gel digested and peptides incubated (4 °C, overnight) with 10 µL of antiphosphorylated tyrosine immunoaffinity beads in immunoprecipitation buffer containing the TLCK trypsin inhibitor (Sigma). Phosphorylated peptides were eluted in 0.1% trifluoroacetic acid (Sigma) and analyzed by mass spectrometry [32].

### 4.5. Western Blotting

Cells were washed in PBS and lysed with ice-cold buffer (50 mmol/L Tris-HCl (pH 8), 150 mmol/L NaCl, 0.5% sodium deoxycholate, 1% NP40, 10% glycerol, 1 mmol/L Na_3_VO_4_, 50 mmol/L NaF, and cOmplete^TM^ Protease Inhibitor Cocktail, Boehringer). Protein concentration was determined with the BCA assay (Interchim, Montluçon, France). Then, 50 µg of proteins were loaded per lane and separated by SDS-PAGE and transferred onto PVDF membranes. Membranes were blocked with 5% BSA in TBS-Tween wash buffer for 1 h, then incubated with the appropriate primary antibody diluted at ≈1 µg/mL in 1% BSA in TBS-Tween. Secondary antibodies were peroxidase-conjugated anti-mouse and anti-rabbit IgG (Jackson ImmunoResearch Laboratories, Suffolk, UK) (diluted 1:5000). Signals were revealed using enhanced chemiluminescence and detected with the G:BOX Chemi system (Syngene).

### 4.6. Immunoprecipitation

Cells were lysed in ice-cold buffer (see above) and ≈700 μg of protein lysates were incubated with 2 μg of antibody followed by incubation with protein-A/G Sepharose beads (Sigma Saint Quentin Fallavier, France) (4 °C, 3 h). Beads were washed four times with ice-cold lysis buffer, and bound proteins were eluted in 2X DTT-containing Laemmli buffer and boiled prior to SDS-PAGE.

### 4.7. Immunofluorescence Staining

Cells were plated on coverslips and transfected with the indicated plasmids. After 24–48 h, cells were washed with PBS, fixed with 3.7% formaldehyde in PBS for 20 min, then permeabilized with 0.1% Triton-X100 at room temperature (RT) for 4 min, or incubated with methanol at –20 °C for 10 min. Cells were incubated with 10 μg/mL of primary antibody (diluted in 0.2% gelatin in TBS) at RT for 2 h. After washes in TBS, coverslips were incubated with FITC- or TRITC- or Cy5-conjugated donkey-anti-mouse or donkey-anti-rabbit secondary antibodies (Jackson ImmunoResearch Laboratories) at RT for 1 h. DNA was stained with Hoechst 33342 (1:10,000; Molecular Probes, Interchim) at RT for 10 min. After mounting with ProLong Gold Antifade reagent (Molecular Probes), images were acquired with a Zeiss Imager M2 microscope with the Apotome system and a Plan Apochromat 40×/1.3 DIC (oil) controlled by the ZEN software (Carl Zeiss Microscopy GmbH), or a Leica DMRA2 microscope (Rueil-Malmaison, France) equipped with an oil immersion ×100/1.4 apochromatic objective and a 12-bit Coolsnap FX CCD camera (Princeton Instruments, Roper Scientific, Evry, France) controlled by the MetaMorph software (Universal Imaging, Roper Scientific). Images were treated with ImageJ (https://imagej.nih.gov/ij/) or Adobe Photoshop (Adobe Sytems, Inc., San Jose, CA).

### 4.8. 2D-Cell Aggregation Assays

First, 10^5^ cells were seeded on 18 mm coverslips in 12-well plates. After a 24 h cell attachment and spreading, cells were incubated with 2 mM MgCl_2_ and 1 mM CaCl_2_ in Hank’s Balanced Salt Solution (HBSS) medium for 15 min. Cell junctions were then disrupted by incubation with 2 mM MgCl_2_ and 4 mM EGTA in HBSS for 2 h. The effect was monitored by E-cadherin staining. Cell junction assembly was restored by switching to DMEM medium containing CaCl_2_. At different times after medium switching (30 to 180 min), cells were processed for immunofluorescence with a FITC-conjugated anti-E-cadherin antibody (36E) and phalloidin-TRITC (Sigma). The length of the E-cadherin signal relative to the total cell border length was determined using ImageJ. Images representative for at least two experiments (more than 100 measurements for randomly selected cell pairs) were selected on each coverslip.

### 4.9. Live-Cell Microscopy

For live cell microscopy, images were acquired using a 20× LD PLAN-NEOFLUAR 0.4 PH2 Korr lens on a Carl Zeiss Axiovert 200 M microscope controlled by the Simple PCI Imaging software (Hamamatsu) and equipped with a full-enclosure environmental chamber heated to 37 °C and an Orca 03GO1 camera (Hamamatsu, Japan). Frames were recorded every 30 min for 50 h. Transfected and nontransfected cells were followed during the same time-lapse using a Ludl motorized XY stage and diascopic illumination shutter. Images were imported as a sequence and the object size changes were analyzed using the Fiji software.

### 4.10. 3D-Cell Aggregation Assays

Cells were completely dissociated by trypsinization, and 12 × 10^4^ cells/well were seeded on ultra-low-attachment surface 24-well plates (Corning), in the presence of 4 mM EGTA or 1 mM CaCl_2_. Plates were then rotated at 80 rpm on a POS-300-Grant-bio rotator in a cell incubator for 18 h. The resulting aggregates were smoothly separated by pipetting, fixed with 0.5% paraformaldehyde for 20 min, and stained with Hoechst 33342. The size and number of aggregates were measured using a Cellomics apparatus (Thermo Scientific) with a Zeiss 20× 0.4 NA Korr LD Plan Neofluar lens and the BioApplications image analysis software.

### 4.11. Wound Healing Assay

Cells were plated in triplicate at 100% confluency on 12-well plates. A confluent area was then scratched with a pipette tip and cell migration into the gap was dynamically followed by video microscopy. Cells were imaged every 30 min for 62 h using a Carl Zeiss Axiovert 200M and a 20× LD PLAN-NEOFLUAR 0.4 PH2 Korr lens, equipped with a full-enclosure environmental 37 °C chamber. Images were acquired using a Photometrics Coolsnap HQ CCD camera controlled by the MetaMorph software. The different cell lines were monitored using a Ludl motorized XY stage and a diascopic illumination shutter. At each time point, the width of the scraped gap was measured in different areas for each scratch. Quantitative analyses were performed with ImageJ.

### 4.12. Cell Migration and Invasion Assays using the xCELLigence Real-Time Cell Analysis (RTCA) Technology

Cell migration and invasion were assessed using the CIM-plates 16 (Roche). Briefly, serum-free medium was added to the lower (160 μL) and upper (50 μL) chamber of each well and plates were allowed to equilibrate for 1 h. Then, 3 × 10^5^ cells in 100 μL serum-free medium were seeded in the upper chamber of which lower side was precoated or not with a thin layer of collagen IV (to monitor invasion or migration, respectively), and incubated at 37 °C for 30 min. Plates were locked in the xCELLigence RTCA DP instrument placed in the incubator for real-time monitoring of cell invasion or migration. In parallel, cell proliferation was evaluated in E-plates (Roche) in which 3000 exponentially growing cells per well were seeded, according to the manufacturer’s instructions.

### 4.13. Matrigel Outgrowth Assay

A 200 μL layer of 5 mg/mL Matrigel (Becton Dickinson) in complete DMEM was deposited at the bottom of 24-well plates and allowed to solidify at 37 °C for 1 h. Then, 2 × 10^4^ cells were slowly diluted in 200 μL of 10 mg/mL Matrigel in cold complete DMEM and carefully deposited on top of the bottom layer. After 30 min at 37 °C, 500 μL of complete DMEM was added to each well, and cells were cultured at 37 °C for 48 h. Images were then acquired using a phase contrast Leica DMIRB inverted microscope and a Photometrics Coolsnap HQ (CCD) camera controlled by the Volocity software. The cell object area and lengths were determined using ImageJ.

### 4.14. Transepithelial Resistance (TER) Measurements

Cells were plated at 100% confluency in 24-well plates in triplicate on polycarbonate membrane (0.4 mm pore size) inserts (6.5 mm diameter; Transwell, Costar). TER measurements were performed using the EVOM Epithelial Volt-Ohm meter (World Precision Instruments Europe, Friedberg, Germany), according to the manufacturer’s protocol.

### 4.15. Animal Experiments

Mice harboring the floxed *Syk* gene (*Syk*^fl/fl^ mice, on a C57BL6 background) were generated for our laboratory at the “Mouse Clinical Institute” (http://www.ics-mci.fr/en/) (ICS, Illkirch, France), and transgenic Wap-Cre C57BL6 mice (strain name: B6.Cg-Tg(Wap-Cre)11738Mam/Nci; strain code: 01XA8) were obtained from the “NCI Mouse repository” (Rockville MD, USA). Recombination mediated by Cre under control of the *Wap* gene promoter is largely restricted to the mammary gland, with higher recombination rates observed at the second pregnancy [49]. Homozygous Wap-Cre;Syk^fl/fl^ conditional *Syk* knockout (*Syk* cKO) mice were generated by breeding these two lines, and the *Syk*^fl/fl^ mice were used as controls. All animal studies were approved by the French Ethics Committee (protocol reference CEEA-LR-12165). Epithelial cells from mouse mammary glands were isolated as described [75]. Using a scalpel, inguinal and abdominal mammary glands were removed, cut into small pieces, and incubated with a collagenase solution (DMEM/F12, 5% fetal calf serum, 2 mg/mL collagenase A (type IV Clostridium histolyticum, Sigma)) with moderate shaking (100 rpm) at 37 °C for 2 h. Digested samples were pelleted and resuspended in 10 mL serum-free medium, pelleted again, and resuspended in 10 mL of DMEM/F12 to remove the collagenase mix and fatty debris. After three short washes (centrifugation at RT for 2 s) and resuspension in 10 mL DMEM/F12 to remove contaminating nonepithelial cells, pellets were resuspended in 6 mL of DNase solution (4 U/mL, Sigma) in DMEM/F12 and incubated at RT for 3 min. The resulting epithelial cell pellets were used for DNA and protein extraction.

### 4.16. DNA Preparation and PCR Analysis

DNA was extracted using the KAPA Mouse Genotyping Kit (Kapa Biosystems, Clinisciences, Nanterre, France) according to the manufacturer’s instructions. For detection of the Wap-Cre transgene, the forward primer W003 (AGCTGTGCCAGCCTCTTC) and the C031 antisense primer (CATCACTCGTTGCATCGACC) were used. For detection of the floxed *Syk* allele, the Ef forward (TGTGACCCAGCATGTGTTTT) and the Er reverse (CATGCATTAGCAGGAAAACCT) primers, or the Lf forward (CGCCCTTGAGGACTGTGTCCA) and Lr reverse (CCCACGGTCTCCCAACACACA) primers were used. The Lf-Er primer combination was used to verify the excision of exon 2 and exon 3 of the *Syk* gene in the mammary glands of Wap-Cre;Syk^fl/fl^ Syk cKO mice.

### 4.17. Histological Staining

Mammary glands were isolated at the indicated developmental stages. Tissues were fixed in 4% paraformaldehyde at 4 °C overnight. For whole-mount staining, mammary glands were washed with PBS, stained with carmine red (20 g/L carmine dye, and 5 g/L aluminum potassium sulfate in distilled water) overnight at RT, destained (2% HCl in 70% ethanol) for 1 h, then dehydrated in increasing concentrations of ethanol for 1 h/each and finally stored in xylene. Images were acquired using a Zeiss Discovery V20 Stereo microscope with an AxioCamMRc CCD camera controlled by the Axiovision software. For immunohistochemistry analysis, paraffin-embedded 3 μm-thick mammary glands sections were deparaffinized and demasked simultaneously with PT Link^®^ (temperature cycle: 65, 95, and 65 °C for 15 min in pH 6 buffer from Dako, Glostrup, Denmark). Immunohistochemistry was performed with an automated Dako stainer. Briefly, endogenous peroxidase activity was blocked by incubation with the Peroxidase Blocking buffer for 5 min. After two washes with PBS/Tween, the nonspecific signal was reduced using a protein blocking reagent (Dako), followed by incubation with the anti-Syk rabbit polyclonal antibody (N19, Santa Cruz Biotechnology) in the provided antibody diluent at RT for 20 min. Sections were then incubated with peroxidase-labeled polymer conjugated to anti-rabbit IgG for 30 min. The peroxidase reaction was carried out using the DAB substrate and sections were counterstained with hematoxylin and mounted with a permanent mounting medium. For hematoxylin and eosin staining, paraffin sections were deparaffinized and stained at the RHEM core facility (IRCM). Images were acquired using a Zeiss Imager M2 microscope and a PlanApochromat 40×/1.3 DIC (oil) objective (Nikon). For immunofluorescence staining, sections were deparaffinized in xylene (for 5 and then for 10 min), rehydrated through an ethanol gradient (100%, 96%, 70% for 5 min each) and then washed in water. For antigen retrieval, sections were incubated in citrate buffer (pH 6) in a water bath at 99 °C for 30 min. After cooling for 20 min, sections were washed with PBS, permeabilized in 0.5% Triton X100/PBS for 30 min, followed by two washes with 0.25% Tween-20/PBS and blocking in 5% goat serum/PBS for 30 min. Sections were then incubated with the primary antibody in 0.5% goat serum/PBS at 4 °C overnight. Following washes with 0.25% Tween-20/PBS, sections were incubated with secondary antibodies in 0.5% goat serum/PBS at RT for 45 min. After several washes, DNA was visualized with Hoechst 33342, and sections were mounted in Moviol solution. Images were acquired using a Zeiss Imager M2 microscope with Apotome using a Plan Apochromat 40×/1.3 DIC (oil) controlled by ZEN (Carl Zeiss Microscopy GmbH).

## 5. Conclusions

Our findings demonstrate that Syk-mediated phosphorylation of the E-cadherin/catenin complex stimulates and maintains mammary epithelial cell integrity thereby preventing tumor cell invasion. These observations contribute to a better understanding of the Syk tumor suppressor activity and its protective role against tumor progression.

## Supplementary Materials

The following are available online at https://www.mdpi.com/2072-6694/11/12/1974/s1, Figure S1a: Mass spectrometric spectra of the GST-E-Cdh and GST-Ctn peptides phosphorylated on different tyrosine residues by GST-Syk in vitro, Figure S1b: Tyrosine phosphorylated E-Cdh and Ctn peptides used for the immunization to generate antibodies that specifically recognize these phosphorylated variants, Figure S2: Catenin phosphorylation is induced by catalytically active DsRed-Syk expression, Figure S3: Transient DsRed-Syk transfection increases E-Cdh signal intensity at intercellular junctions, Figure S4: Oxidative stress increases E-Cdh phosphorylation at adherens junctions, Figure S5: GFP-Syk expression promotes 2D cell aggregation, Figure S6: Validation of Syk knockout in Wap-Cre;Syk^fl/fl^ mice, Figure S7: Syk is expressed in luminal epithelial cells of the mammary gland, Figure S8: Syk loss negatively affects epithelial morphogenesis in involuting mammary glands.
