# Supplementary material for: The Syk Kinase Promotes Mammary Epithelial Integrity and Inhibits Breast Cancer Invasion by Stabilizing the E-Cadherin/Catenin Complex
Source: Cancers (Basel). 2019 Dec 7;11(12):1974. doi: 10.3390/cancers11121974 (PMC6966528; doi:10.3390/cancers11121974)
Supplement: Supplementary file 1 [file cancers-11-01974-s001.zip › cancers-649365 whole blot Figs Cancers.pdf]

Figure 1 (a)

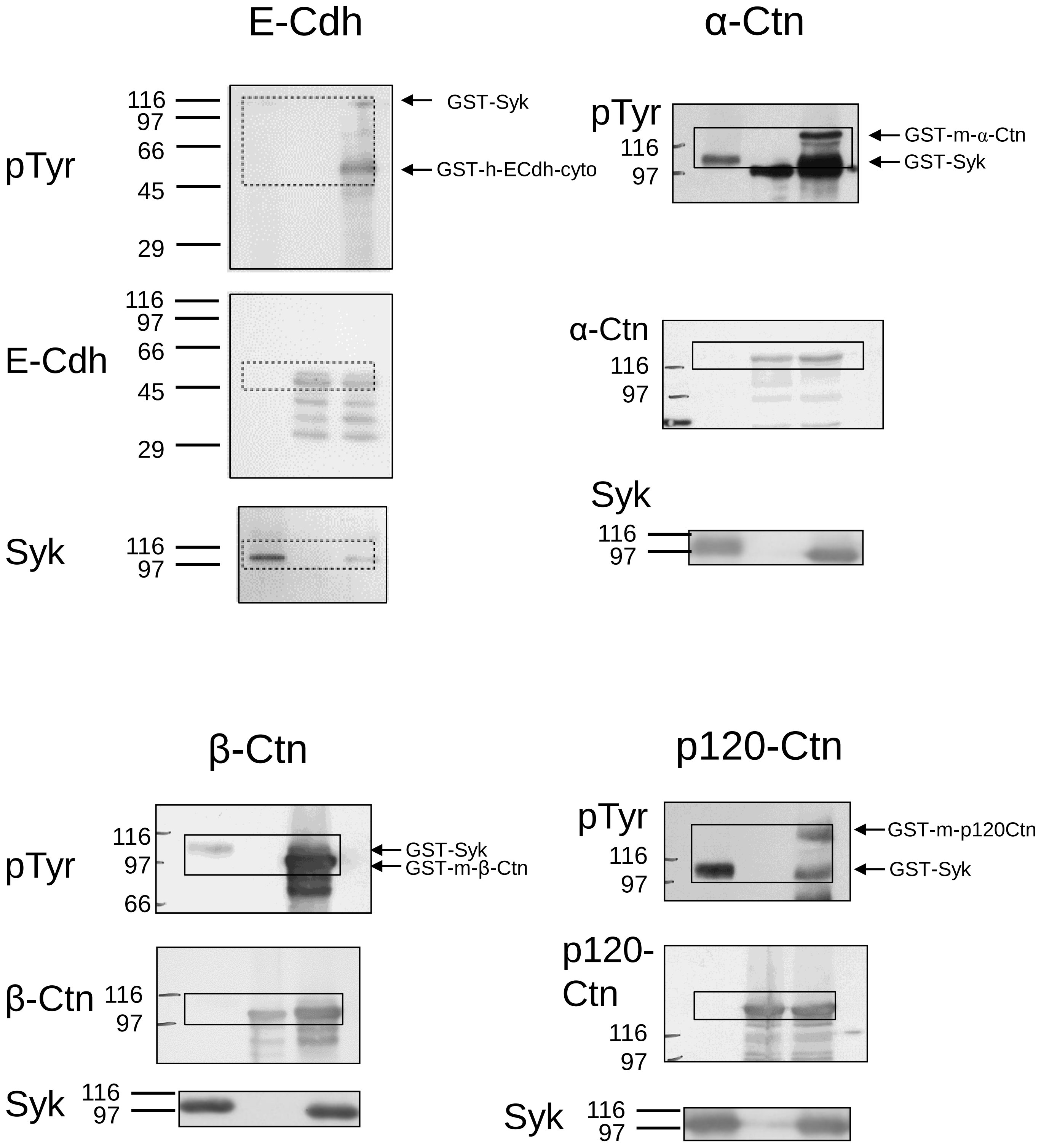
E-Cdh
α-Ctn
pTyr
116
97
66
45
29
GST-Syk
GST-h-ECdh-cyto
GST-m-α-Ctn
E-Cdh
α-Ctn
Syk
β-Ctn
p120-Ctn
GST-m-β-Ctn
GST-m-p120Ctn
p120-
Ctn

Figure 2(a)

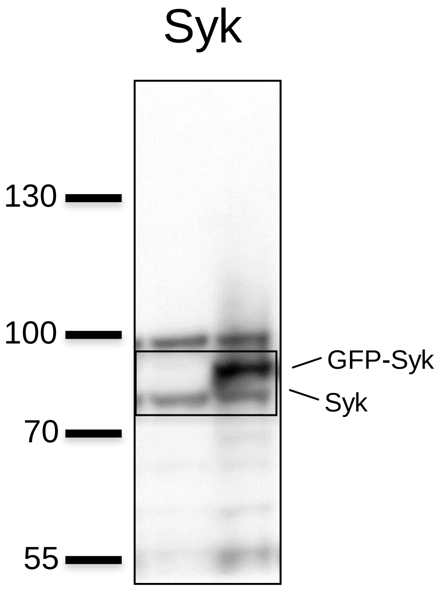

Syk
130
100
70
55
GFP-Syk
Syk

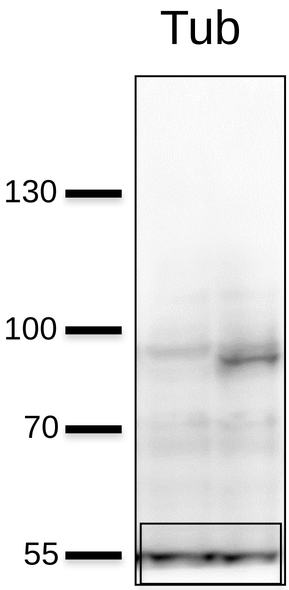

Tub
130
100
70
55

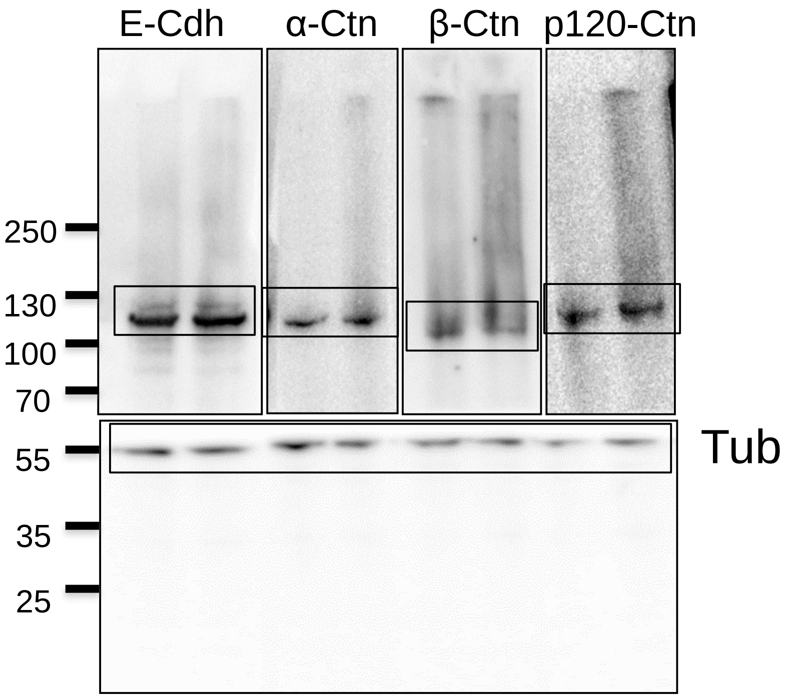

E-Cdh
α-Ctn
β-Ctn
p120-Ctn
250
130
100
70
55
35
25
Tub

Figure 2(b)

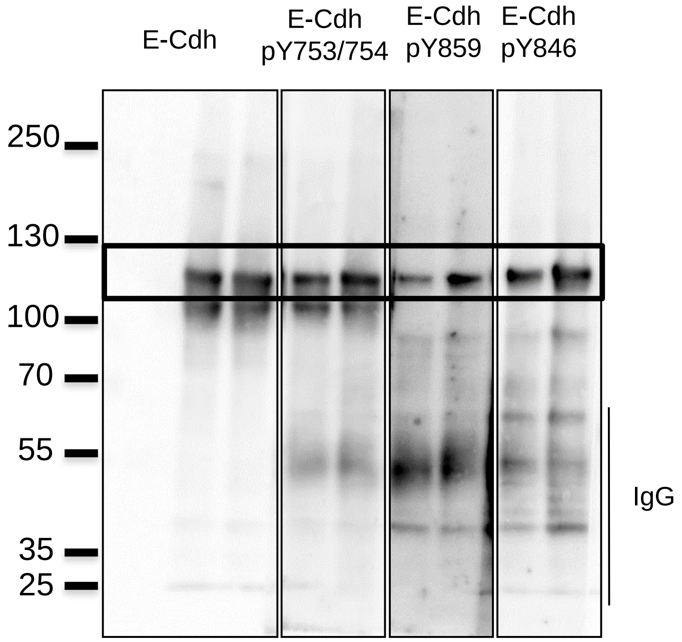
E-Cdh
E-Cdh pY753/754
E-Cdh pY859
E-Cdh pY846
250
130
100
70
55
35
25
IgG

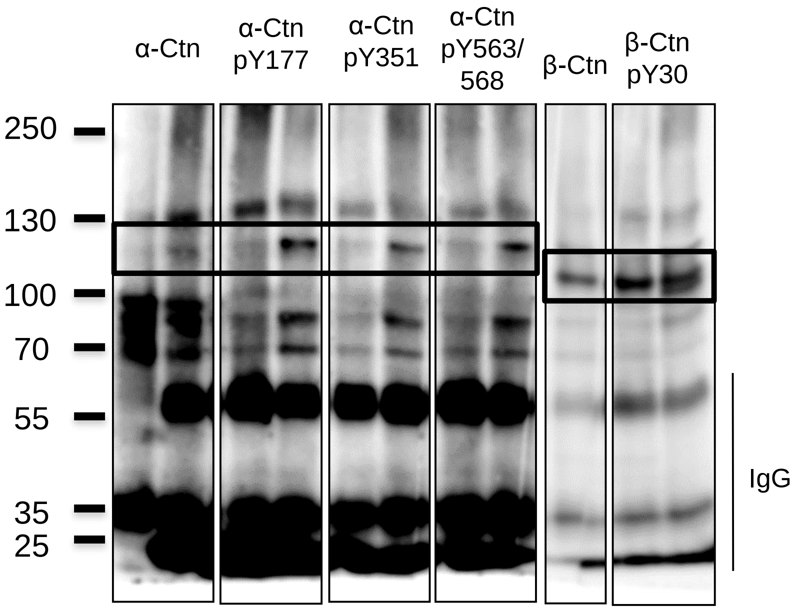
α-Ctn
α-Ctn pY177
α-Ctn pY351
α-Ctn pY563/ 568
β-Ctn
β-Ctn pY30
250
130
100
70
55
35
25
IgG

Figure 2(c)

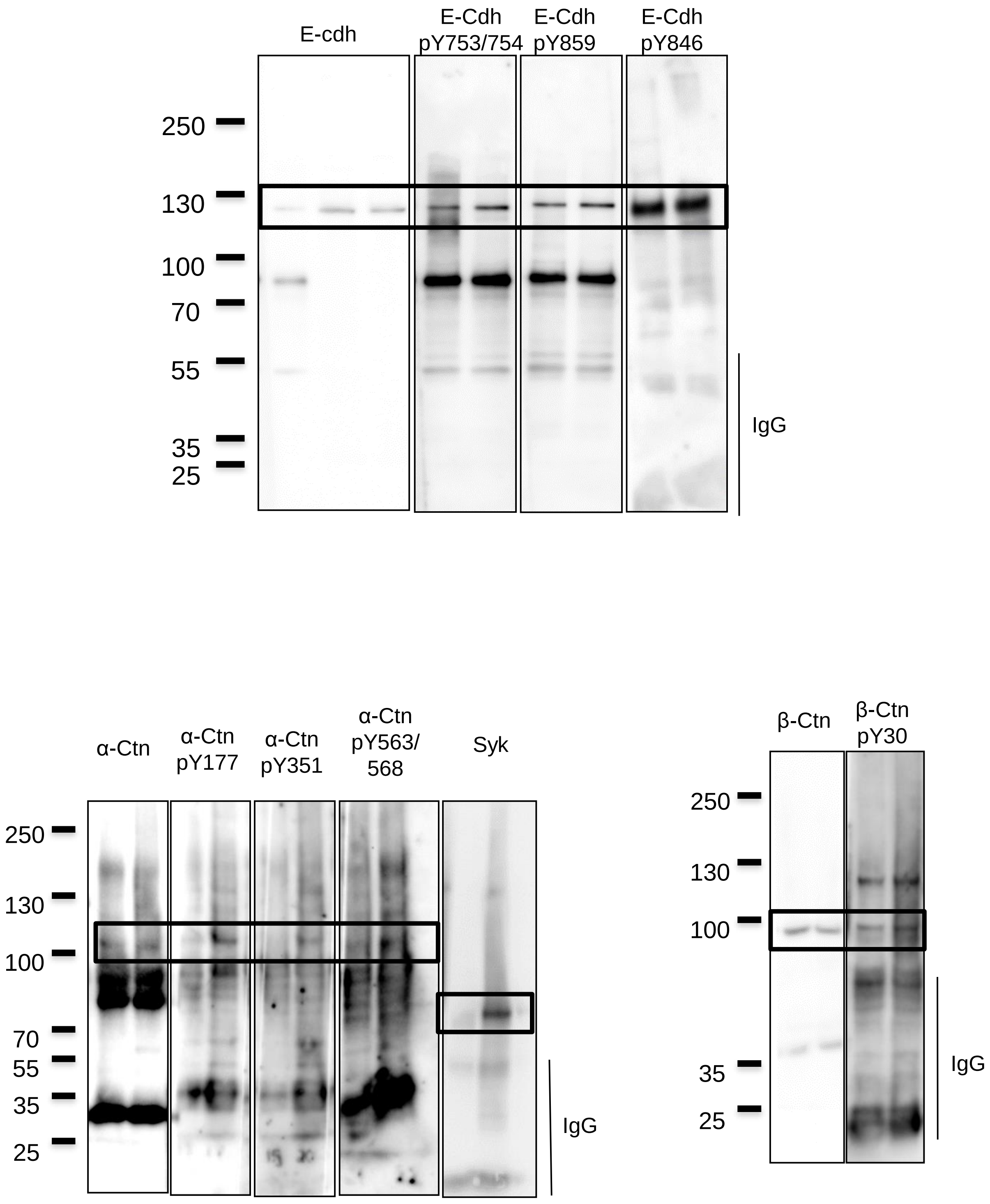
E-cdh
E-Cdh pY753/754
E-Cdh pY859
E-Cdh pY846
250
130
100
70
55
35
25
IgG
α-Ctn
α-Ctn pY177
α-Ctn pY351
α-Ctn pY563/568
Syk
250
130
100
70
55
35
25
IgG
β-Ctn
β-Ctn pY30
250
130
100
35
25
IgG

Figure 2(d)

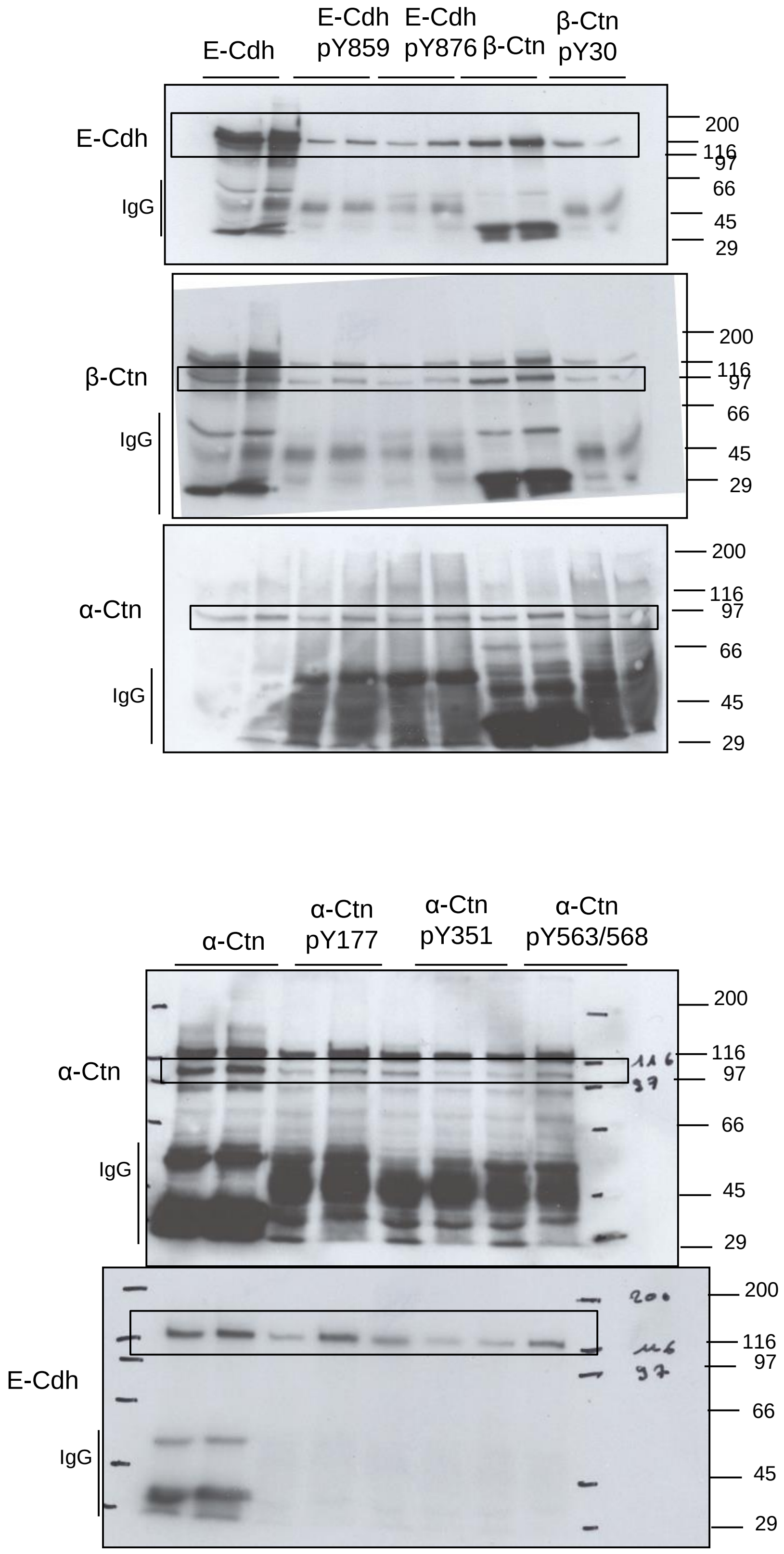
E-Cdh
E-Cdh pY859
E-Cdh pY876
β-Ctn
β-Ctn pY30
E-Cdh
IgG
β-Ctn
IgG
α-Ctn
IgG
200
116
97
66
45
29
α-Ctn
α-Ctn pY177
α-Ctn pY351
α-Ctn pY563/568
α-Ctn
IgG
E-Cdh
IgG

Figure 2(e,f)

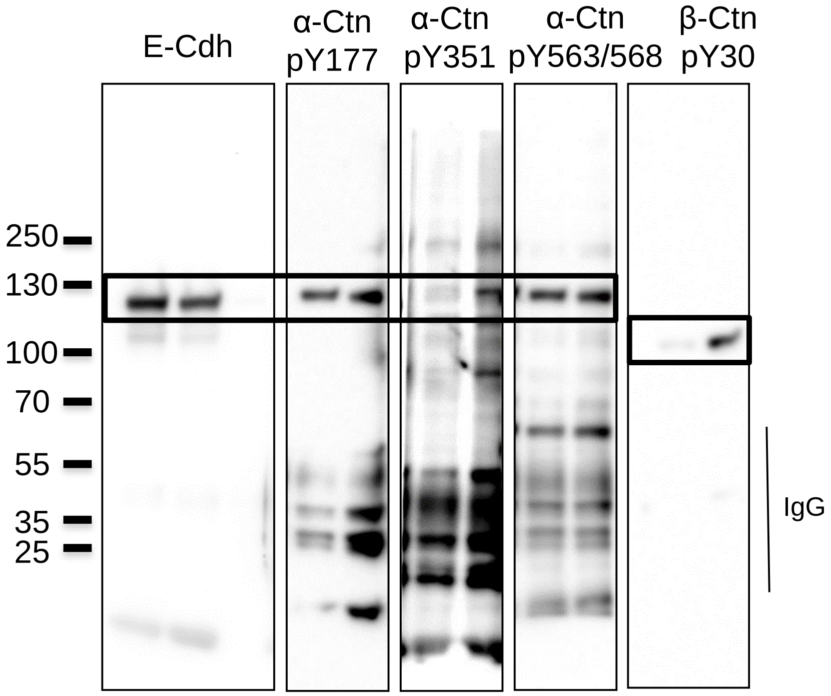
E-Cdh
α-Ctn pY177
α-Ctn pY351
α-Ctn pY563/568
β-Ctn pY30
250
130
100
70
55
35
25
IgG

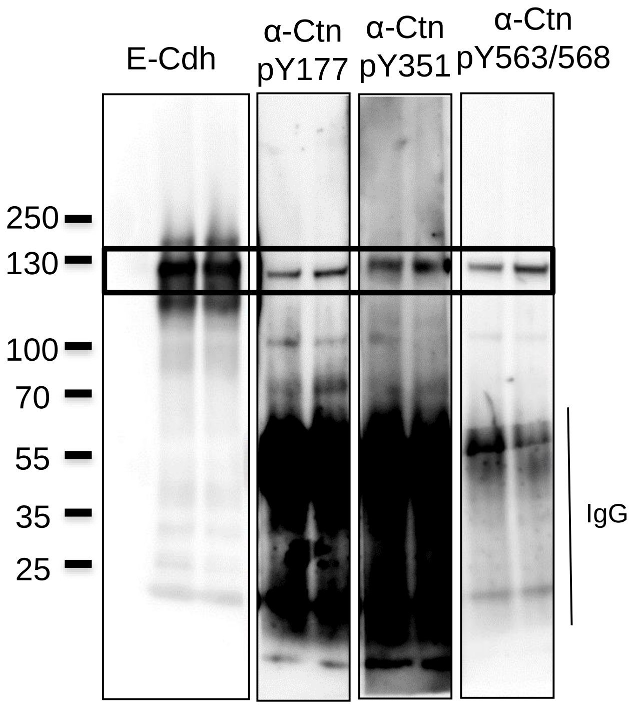
E-Cdh
α-Ctn pY177
α-Ctn pY351
α-Ctn pY563/568
250
130
100
70
55
35
25
IgG

Figure 3 (a)

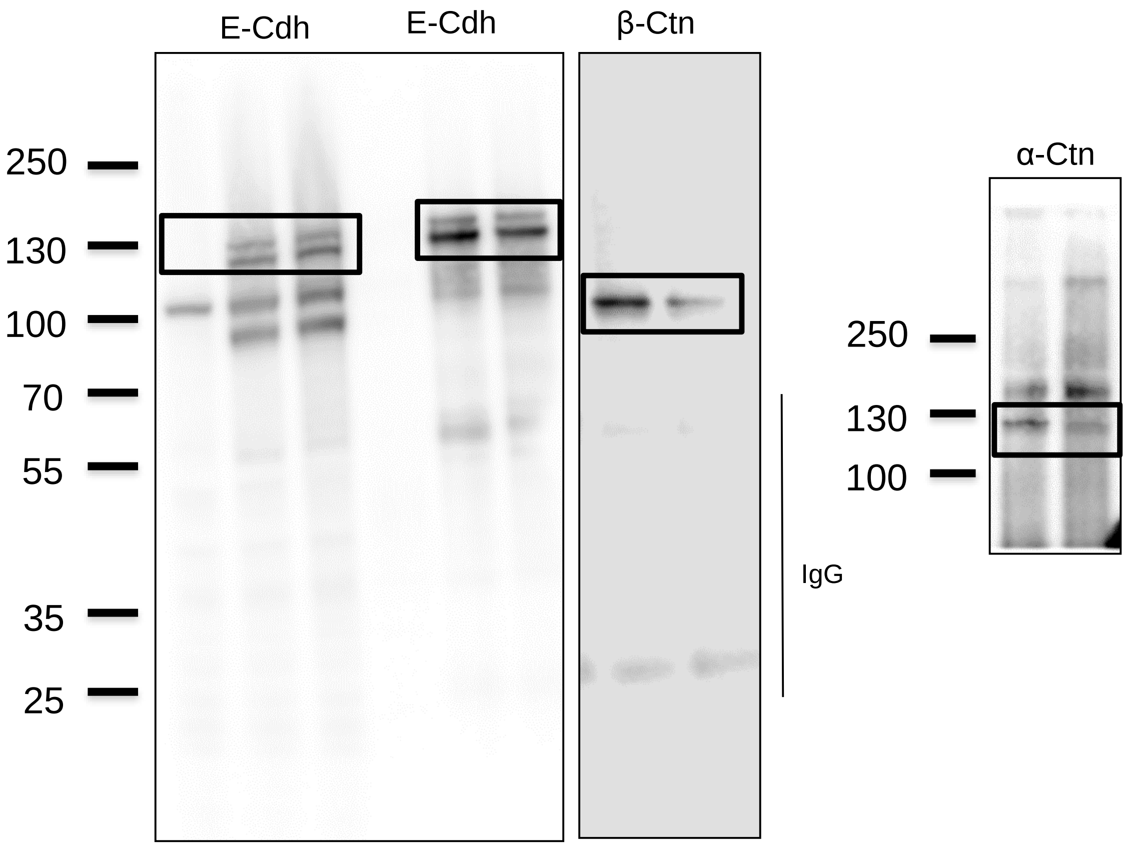

Figure 3 (d,e,f)

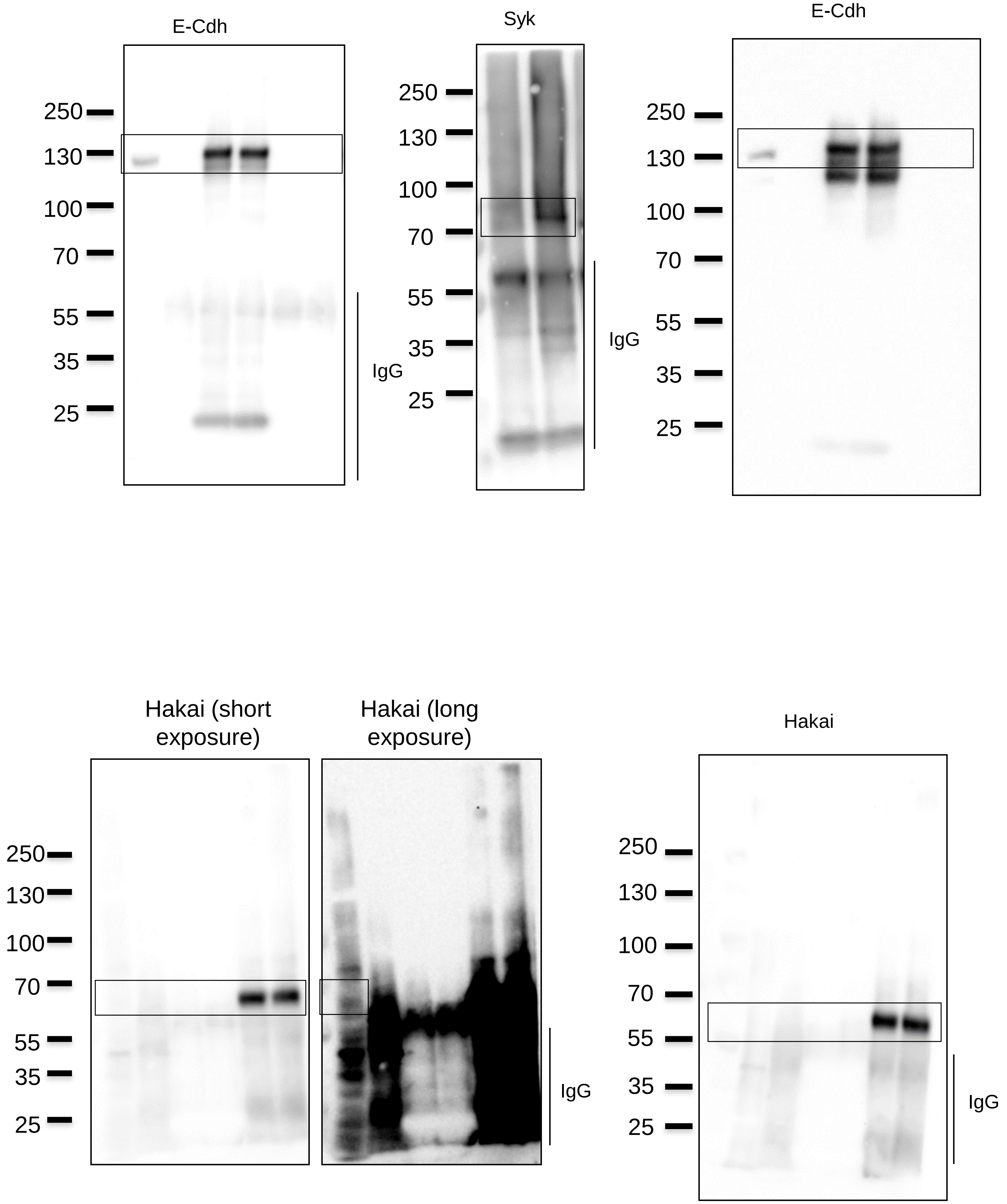
E-Cdh
250
130
100
70
55
35
25
IgG
Syk
250
130
100
70
55
35
25
IgG
E-Cdh
250
130
100
70
55
35
25
Hakai (short exposure)
Hakai (long exposure)
250
130
100
70
55
35
25
IgG
Hakai
250
130
100
70
55
35
25
IgG

Figure 4(e)

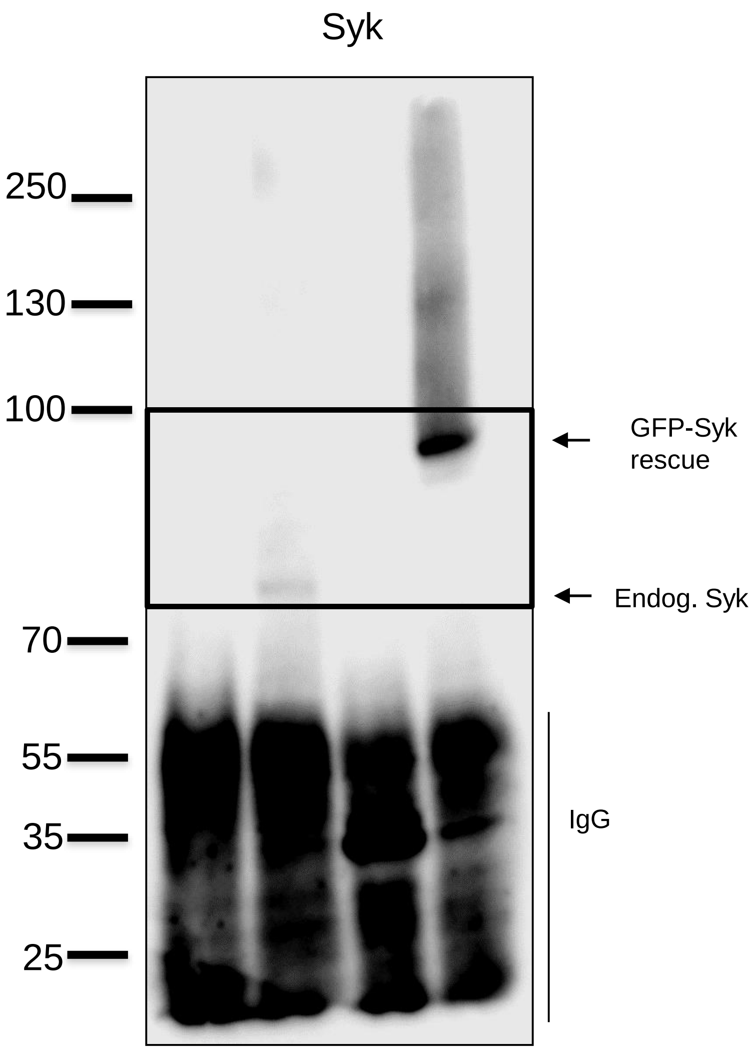

Figure 5(b)

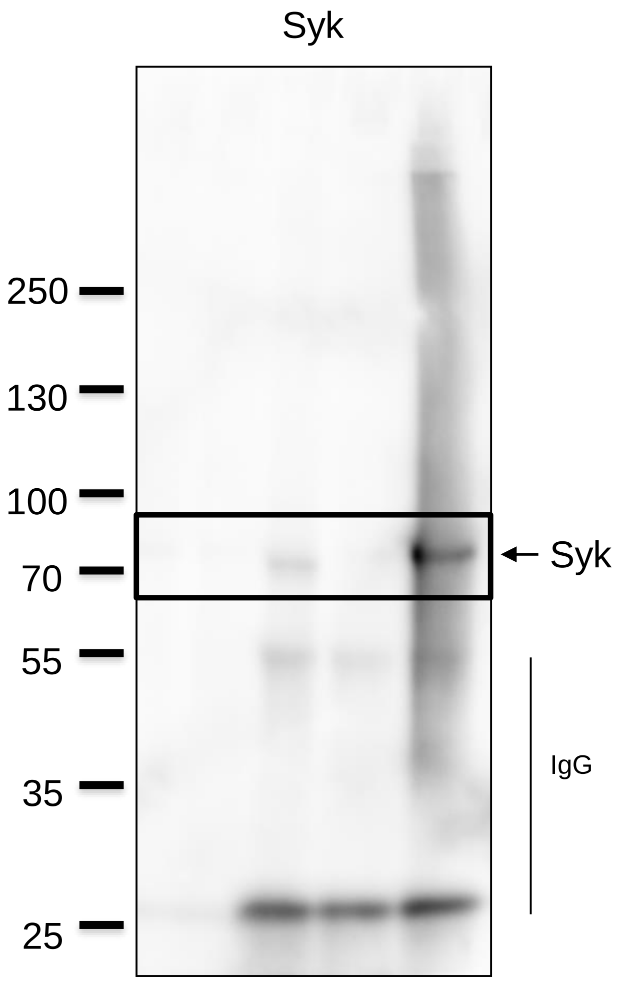

Figure 6(a, part 1)

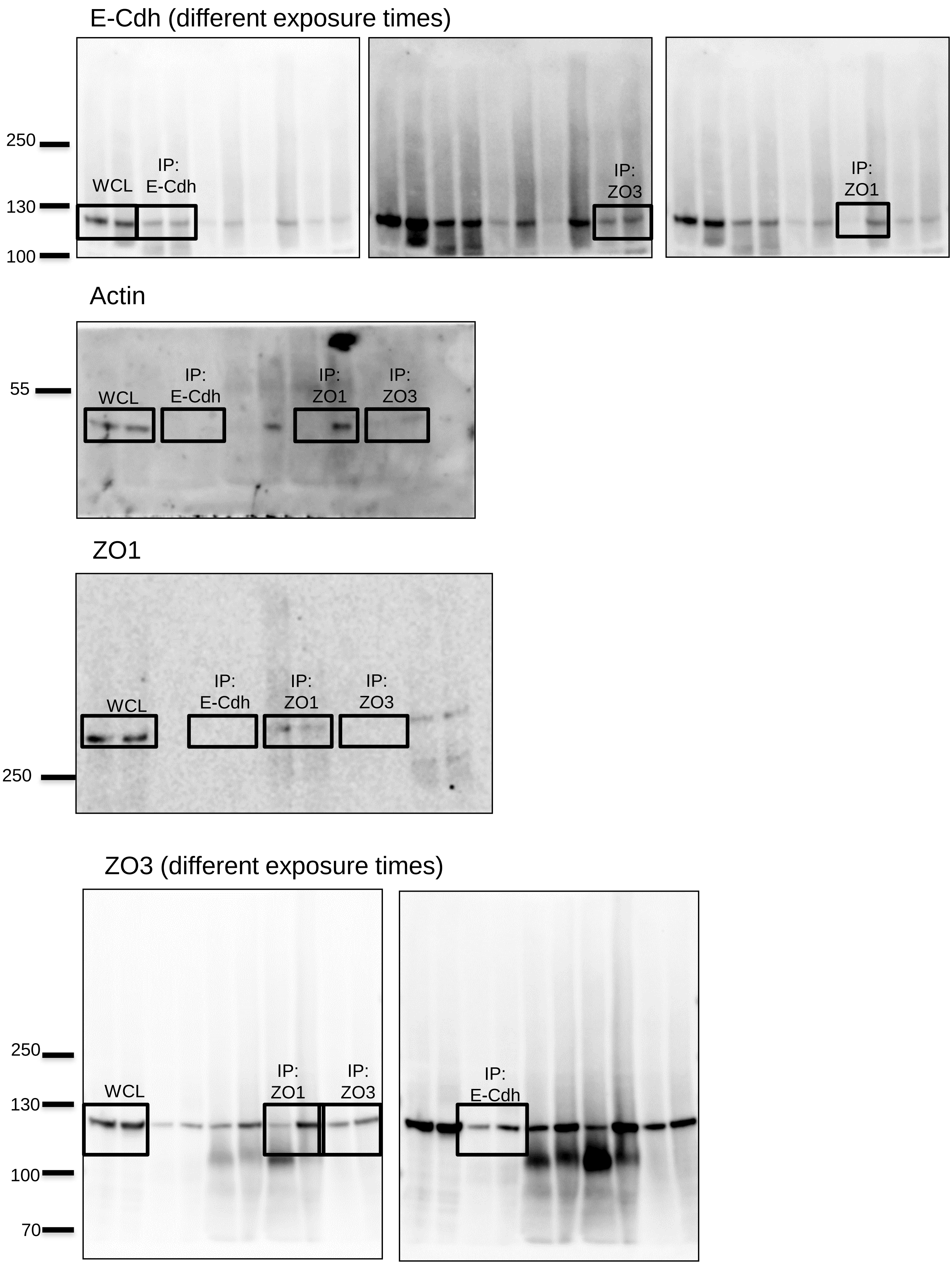

Figure 6(a, part 2)

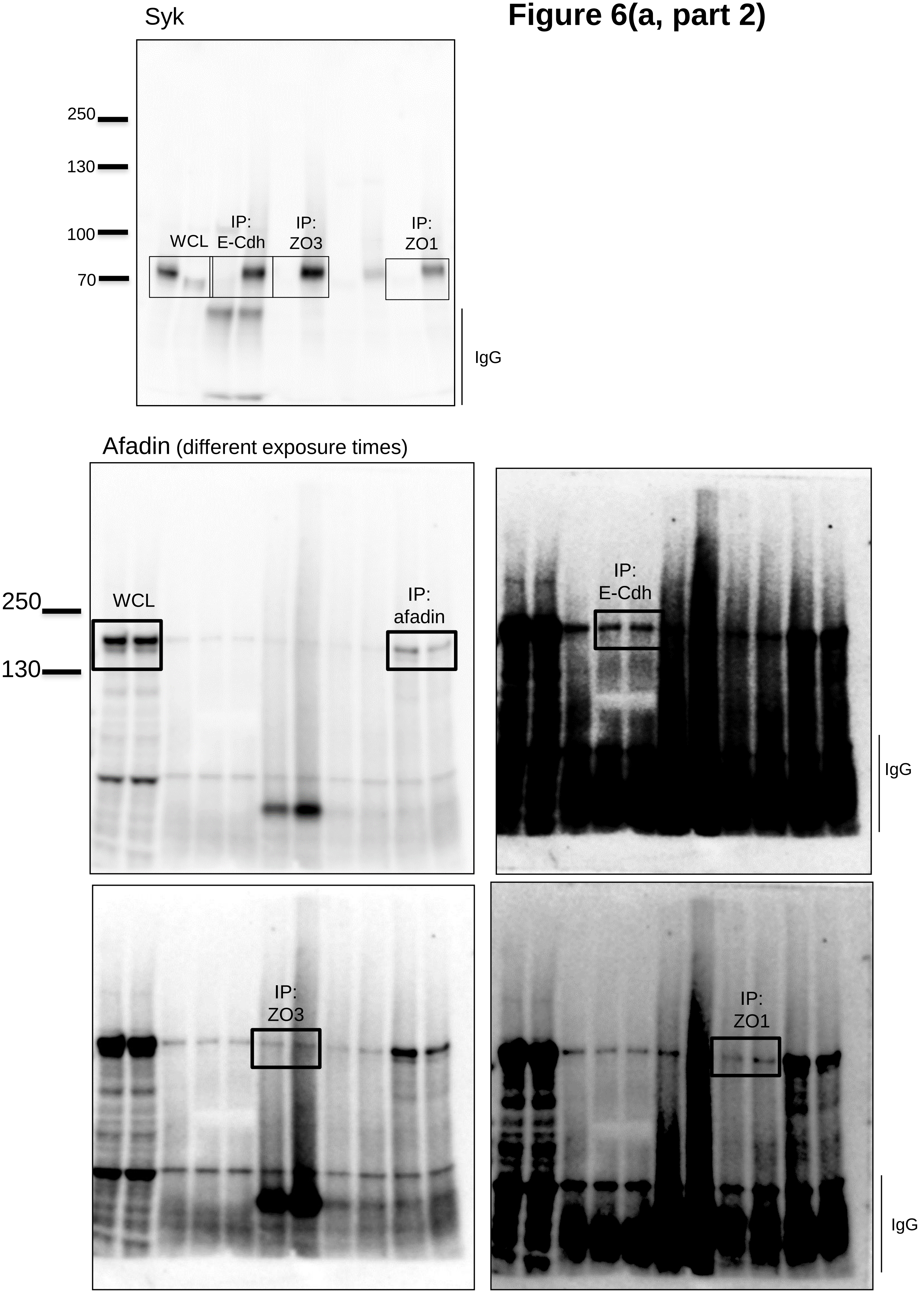

Figure 7(c)

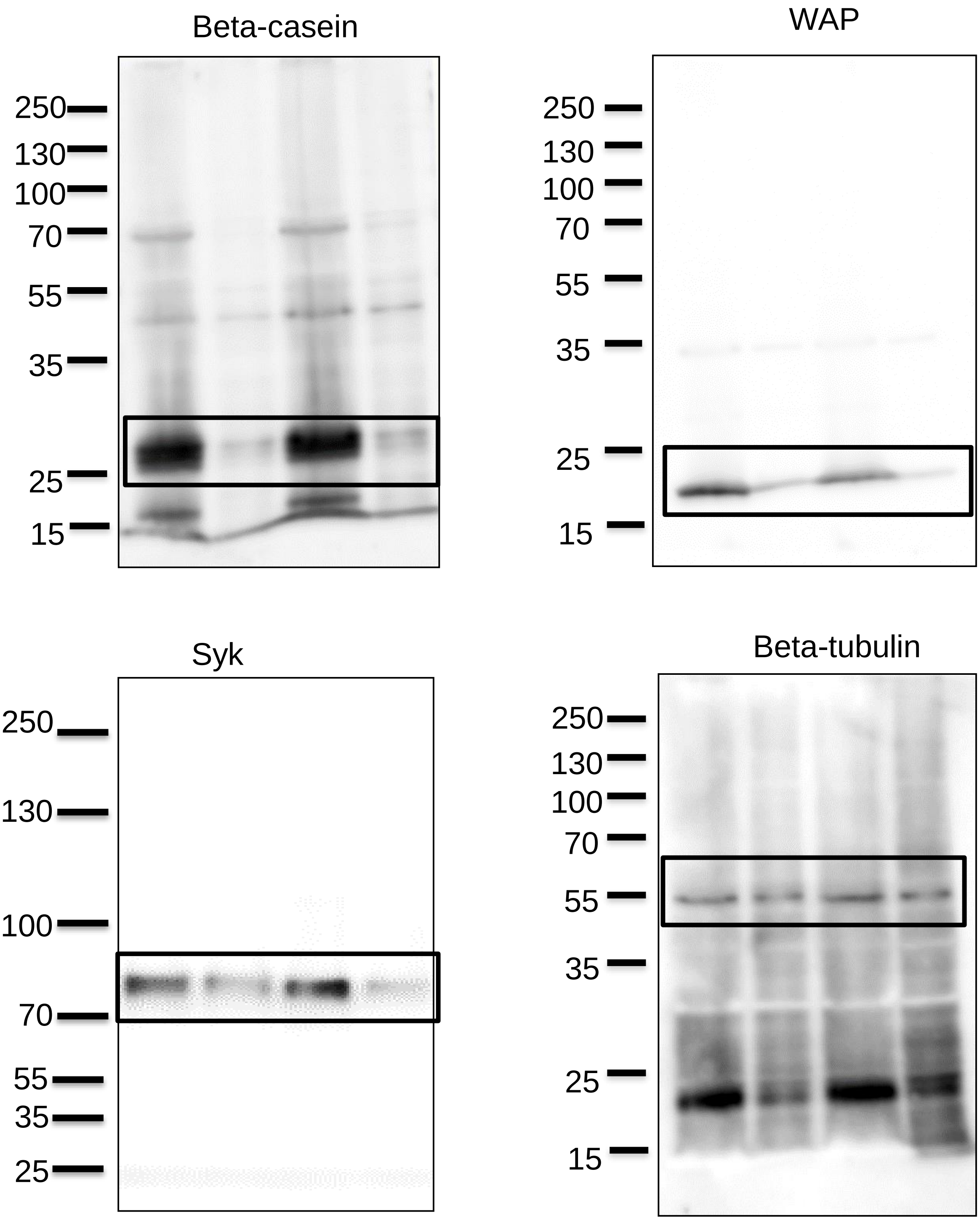
Beta-casein
250
130
100
70
55
35
25
15
WAP
250
130
100
70
55
35
25
15
Syk
250
130
100
70
55
35
25
Beta-tubulin
250
130
100
70
55
35
25
15
