# Supplementary material for: The Syk Kinase Promotes Mammary Epithelial Integrity and Inhibits Breast Cancer Invasion by Stabilizing the E-Cadherin/Catenin Complex
Source: Cancers (Basel). 2019 Dec 7;11(12):1974. doi: 10.3390/cancers11121974 (PMC6966528; doi:10.3390/cancers11121974)
Supplement: Supplementary file 1 [file cancers-11-01974-s001.zip › cancers-649395-supplementary.pdf]

Kassouf et al.

Suppl Figure S1 (a)

Mass spectrometric spectra of the GST-E-Cdh and GST-Ctn peptides phosphorylated on different tyrosine residues by GST-Syk in vitro.

#1. E-cadherin pTyr 753
#2. E-cadherin pTyr 754
#3. E-cadherin pTyr 859
#4. E-cadherin pTyr 876
#5. α-catenin pTyr 177
#6. α-catenin pTyr 351
7. α-catenin pTyr 563 and 568
8. β-catenin pTyr 30

MS2 Spectrum #1. E-cadherine pTyr 753.

MS/MS Fragmentation of **DNVpY753YYDEEGGGEEDQDFDLSQLHR**
Found in **CADH1_HUMAN**, P12830|CADH1_HUMAN Epithelial cadherin precursor - Homo sapiens (Human)
Match to Query 626: 3072.262386 from (1025.094738,3+)

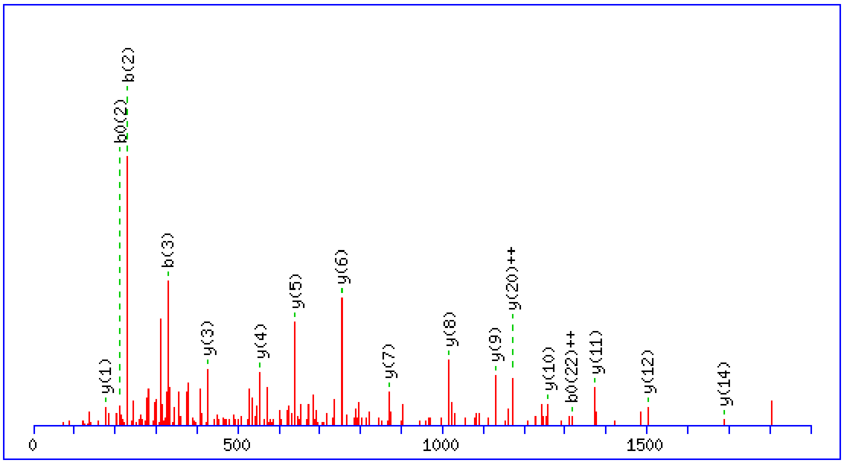

Variable modifications:
Y4 : Phospho (Y)
Ions Score: 87 Expect: 7.8e-08
Matches (Bold Red): 17/278 fragment ions using 20 most intense peaks

#	b	b++	b*	b*++	b0	b0++	Seq.	y	y++	y*	y*++	y0	y0++	[illegible]
1	116.03	58.52			98.02	49.52	D							[illegible]
2	**230.08**	115.54	213.05	107.03	**212.07**	106.54	N	2958.17	1479.59	2941.15	1471.08	2940.16	1470.59	[illegible]
3	**329.15**	165.08	312.12	156.56	311.13	156.07	V	2844.13	1422.57	2827.10	1414.06	2826.12	1413.56	[illegible]
4	492.21	246.61	475.18	238.09	474.20	237.60	Y	2745.06	1373.03	2728.04	1364.52	2727.05	1364.03	[illegible]
5	735.24	368.12	718.21	359.61	717.23	359.12	Y	2582.00	1291.50	2564.97	1282.99	2563.99	1282.50	[illegible]
6	898.30	449.65	881.28	441.14	880.29	440.65	Y	2338.97	**1169.99**	2321.94	1161.48	2320.96	1160.98	[illegible]
7	1013.33	507.17	996.30	498.65	995.32	498.16	D	2175.91	1088.46	2158.88	1079.94	2157.90	1079.45	[illegible]
8	1142.37	571.69	1125.34	563.18	1124.36	562.68	E	2060.88	1030.94	2043.85	1022.43	2042.87	1021.94	[illegible]
9	1271.41	636.21	1254.39	627.70	1253.40	627.21	E	1931.84	966.42	1914.81	957.91	1913.83	957.42	[illegible]
10	1328.44	664.72	1311.41	656.21	1310.42	655.72	G	1802.79	901.90	1785.77	893.39	1784.78	892.90	[illegible]
11	1385.46	693.23	1368.43	684.72	1367.45	684.23	G	1745.77	873.39	1728.75	864.88	1727.76	864.38	[illegible]
12	1442.48	721.74	1425.45	713.23	1424.47	712.74	G	**1688.75**	844.88	1671.72	836.37	1670.74	835.87	[illegible]
13	1571.52	786.26	1554.49	777.75	1553.51	777.26	E	1631.73	816.37	1614.70	807.86	1613.72	807.36	[illegible]
14	1700.56	850.79	1683.54	842.27	1682.55	841.78	E	**1502.69**	751.85	1485.66	743.33	1484.68	742.84	[illegible]
15	1815.59	908.30	1798.56	899.79	1797.58	899.29	D	**1373.64**	687.33	1356.62	678.81	1355.63	678.32	[illegible]
16	1943.65	972.33	1926.62	963.81	1925.64	963.32	Q	**1258.62**	629.81	1241.59	621.30	1240.61	620.81	[illegible]
17	2058.68	1029.84	2041.65	1021.33	2040.67	1020.84	D	**1130.56**	565.78	1113.53	557.27	1112.55	556.78	
18	2205.74	1103.38	2188.72	1094.86	2187.73	1094.37	F	**1015.53**	508.27	998.51	499.76	997.52	499.26	
19	2320.77	1160.89	2303.74	1152.38	2302.76	1151.88	D	**868.46**	434.74	851.44	426.22	850.45	425.73	
20	2433.86	1217.43	2416.83	1208.92	2415.84	1208.43	L	**753.44**	377.22	736.41	368.71	735.43	368.22	
21	2520.89	1260.95	2503.86	1252.43	2502.88	1251.94	S	**640.35**	320.68	623.33	312.17	622.34	311.67	
22	2648.95	1324.98	2631.92	1316.46	2630.94	**1315.97**	Q	**553.32**	277.16	536.29	268.65			
23	2762.03	1381.52	2745.00	1373.01	2744.02	1372.51	L	**425.26**	213.13	408.24	204.62			
24	2899.09	1450.05	2882.06	1441.53	2881.08	1441.04	H	312.18	156.59	295.15	148.08			
25							R	**175.12**	88.06	158.09	79.55			

MS2 Spectrum #2. E-cadherine pTyr 754.

MS/MS Fragmentation of **DNVYpY[754]YDEEGGGEEDQDFDLSQLHR**
Found in **CADH1_HUMAN**, P12830|CADH1_HUMAN Epithelial cadherin precursor - Homo sapiens (Human)
Match to Query 626: 3072.262386 from (1025.094738,3+)

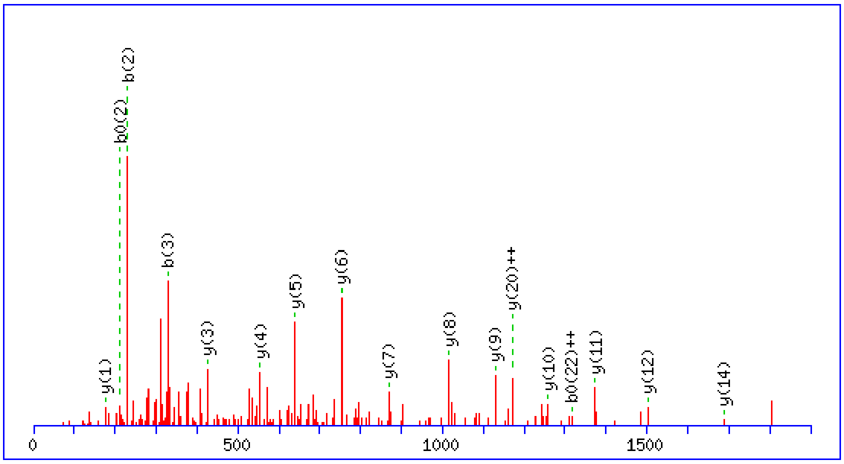

Variable modifications:
Y5 : Phospho (Y)
Ions Score: 87 Expect: 7.8e-08
Matches (Bold Red): 17/278 fragment ions using 20 most intense peaks

#	b	b++	b*	b*++	b0	b0++	Seq.	y	y++	y*	y*++	y0	y0++	[illegible]
1	116.03	58.52			98.02	49.52	D							[illegible]
2	230.08	115.54	213.05	107.03	212.07	106.54	N	2958.17	1479.59	2941.15	1471.08	2940.16	1470.59	[illegible]
3	329.15	165.08	312.12	156.56	311.13	156.07	V	2844.13	1422.57	2827.10	1414.06	2826.12	1413.56	[illegible]
4	492.21	246.61	475.18	238.09	474.20	237.60	Y	2745.06	1373.03	2728.04	1364.52	2727.05	1364.03	[illegible]
5	735.24	368.12	718.21	359.61	717.23	359.12	Y	2582.00	1291.50	2564.97	1282.99	2563.99	1282.50	[illegible]
6	898.30	449.65	881.28	441.14	880.29	440.65	Y	2338.97	1169.99	2321.94	1161.48	2320.96	1160.98	[illegible]
7	1013.33	507.17	996.30	498.65	995.32	498.16	D	2175.91	1088.46	2158.88	1079.94	2157.90	1079.45	[illegible]
8	1142.37	571.69	1125.34	563.18	1124.36	562.68	E	2060.88	1030.94	2043.85	1022.43	2042.87	1021.94	[illegible]
9	1271.41	636.21	1254.39	627.70	1253.40	627.21	E	1931.84	966.42	1914.81	957.91	1913.83	957.42	[illegible]
10	1328.44	664.72	1311.41	656.21	1310.42	655.72	G	1802.79	901.90	1785.77	893.39	1784.78	892.90	[illegible]
11	1385.46	693.23	1368.43	684.72	1367.45	684.23	G	1745.77	873.39	1728.75	864.88	1727.76	864.38	[illegible]
12	1442.48	721.74	1425.45	713.23	1424.47	712.74	G	1688.75	844.88	1671.72	836.37	1670.74	835.87	[illegible]
13	1571.52	786.26	1554.49	777.75	1553.51	777.26	E	1631.73	816.37	1614.70	807.86	1613.72	807.36	[illegible]
14	1700.56	850.79	1683.54	842.27	1682.55	841.78	E	1502.69	751.85	1485.66	743.33	1484.68	742.84	[illegible]
15	1815.59	908.30	1798.56	899.79	1797.58	899.29	D	1373.64	687.33	1356.62	678.81	1355.63	678.32	[illegible]
16	1943.65	972.33	1926.62	963.81	1925.64	963.32	Q	1258.62	629.81	1241.59	621.30	1240.61	620.81	[illegible]
17	2058.68	1029.84	2041.65	1021.33	2040.67	1020.84	D	1130.56	565.78	1113.53	557.27	1112.55	556.78	
18	2205.74	1103.38	2188.72	1094.86	2187.73	1094.37	F	1015.53	508.27	998.51	499.76	997.52	499.26	
19	2320.77	1160.89	2303.74	1152.38	2302.76	1151.88	D	868.46	434.74	851.44	426.22	850.45	425.73	
20	2433.86	1217.43	2416.83	1208.92	2415.84	1208.43	L	753.44	377.22	736.41	368.71	735.43	368.22	
21	2520.89	1260.95	2503.86	1252.43	2502.88	1251.94	S	640.35	320.68	623.33	312.17	622.34	311.67	
22	2648.95	1324.98	2631.92	1316.46	2630.94	1315.97	Q	553.32	277.16	536.29	268.65			
23	2762.03	1381.52	2745.00	1373.01	2744.02	1372.51	L	425.26	213.13	408.24	204.62			
24	2899.09	1450.05	2882.06	1441.53	2881.08	1441.04	H	312.18	156.59	295.15	148.08			
25							R	175.12	88.06	158.09	79.55			

MS2 Spectrum #3. E-cadherine pTyr 859.

MS/MS Fragmentation of **DQDpY^{859}DYLNEWGNR**
Found in **CADH1_HUMAN**, P12830|CADH1_HUMAN Epithelial cadherin precursor - Homo sapiens (Human)
Match to Query 521: 1766.564278 from(884.289415,2+)

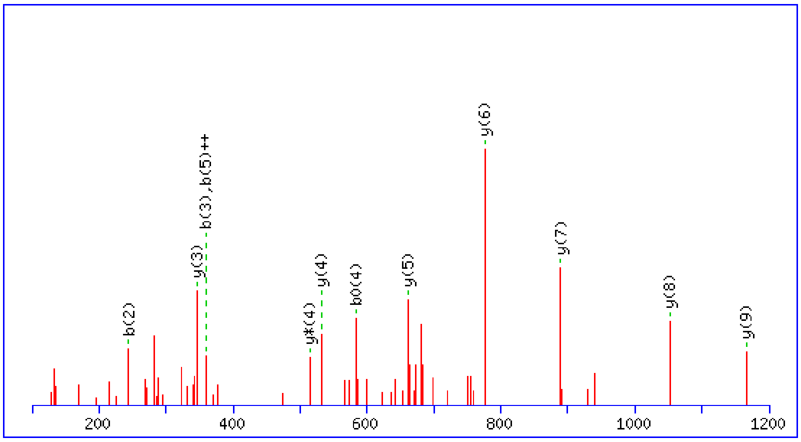

Variable modifications:
Y4 : Phospho (Y)
Ions Score: 39 Expect: 0.0049
Matches (Bold Red): 12/134 fragment ions using 15 most intense peaks

#	b	b^{++}	b^{*}	b^{*++}	b^{0}	b^{0++}	Seq.	y	y^{++}	y^{*}	y^{*++}	y^{0}	y^{0++}	#
1	116.03	58.52			98.02	49.52	D							13
2	**244.09**	122.55	227.07	114.04	226.08	113.54	Q	1652.64	826.82	1635.61	818.31	1634.63	817.82	12
3	**359.12**	180.06	342.09	171.55	341.11	171.06	D	1524.58	762.79	1507.55	754.28	1506.57	753.79	11
4	602.15	301.58	585.12	293.07	**584.14**	292.57	Y	1409.55	705.28	1392.53	696.77	1391.54	696.27	10
5	717.18	**359.09**	700.15	350.58	699.17	350.09	D	**1166.52**	583.76	1149.50	575.25	1148.51	574.76	9
6	880.24	440.62	863.21	432.11	862.23	431.62	Y	**1051.50**	526.25	1034.47	517.74	1033.49	517.25	8
7	993.32	497.17	976.30	488.65	975.31	488.16	L	**888.43**	444.72	871.41	436.21	870.42	435.71	7
8	1107.37	554.19	1090.34	545.67	1089.36	545.18	N	**775.35**	388.18	758.32	379.66	757.34	379.17	6
9	1236.41	618.71	1219.38	610.19	1218.40	609.70	E	**661.31**	331.16	644.28	322.64	643.29	322.15	5
10	1422.49	711.75	1405.46	703.23	1404.48	702.74	W	**532.26**	266.63	**515.24**	258.12			4
11	1479.51	740.26	1462.48	731.75	1461.50	731.25	G	**346.18**	173.60	329.16	165.08			3
12	1593.55	797.28	1576.53	788.77	1575.54	788.27	N	289.16	145.08	272.14	136.57			2
13							R	175.12	88.06	158.09	79.55			1

MS2 Spectrum #4. E-cadherine pTyr 876.

MS/MS Fragmentation of **LADMpY^{876}GGGEDD**
Found in **CADH1_HUMAN**, P12830|CADH1_HUMAN Epithelial cadherin precursor - Homo sapiens (Human)
Match to Query 790: 1237.409452 from(619.712002,2+)

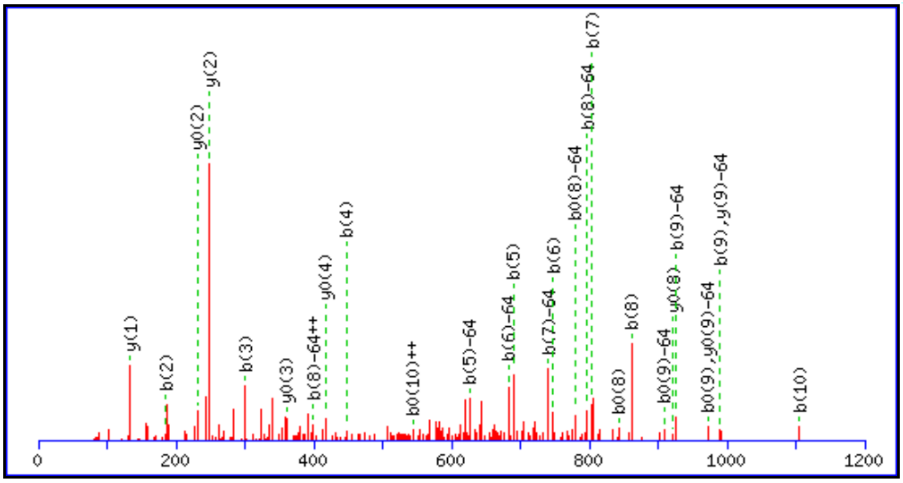

Variable modifications:
M4 : Oxidation (M), with neutral losses 0.00(shown in table), 64.00
Y5 : Phospho (Y)
Ions Score: 28 Expect: 0.27
Matches (Bold Red): 28/116 fragment ions using 59 most intense peaks

#	b	b++	b0	b0++	Seq.	y	y++	y0	y0++	#
1	114,09	57,55			L					11
2	**185,13**	93,07			A	1125,31	563,16	1107,3	554,15	10
3	**300,16**	150,58	282,14	141,58	D	1054,27	527,64	1036,26	518,63	9
4	**447,19**	224,1	429,18	215,09	M	939,24	470,13	**921,23**	461,12	8
5	**690,22**	345,61	672,21	336,61	Y	792,21	396,61	774,2	387,6	7
6	**747,24**	374,12	729,23	365,12	G	549,18	275,09	531,17	266,09	6
7	**804,26**	402,64	786,25	393,63	G	492,16	246,58	474,15	237,58	5
8	**861,28**	431,15	**843,27**	422,14	G	435,14	218,07	**417,13**	209,07	4
9	**990,33**	495,67	**972,32**	486,66	E	378,11	189,56	**360,1**	180,56	3
10	**1105,35**	553,18	1087,34	**544,18**	D	**249,07**	125,04	**231,06**	116,03	2
11					D	**134,04**	67,53	116,03	58,52	1

MS2 Spectrum #5. α-catenin pTyr 177

MS/MS Fragmentation of **NAGNEQDLGIQpY^{177}K**
Found in **CTNA1_HUMAN**, P35221|CTNA1_HUMAN Catenin alpha-1
Homo sapiens (Human)
Match to Query 573: 1528.589108 from (765.301830,2+)

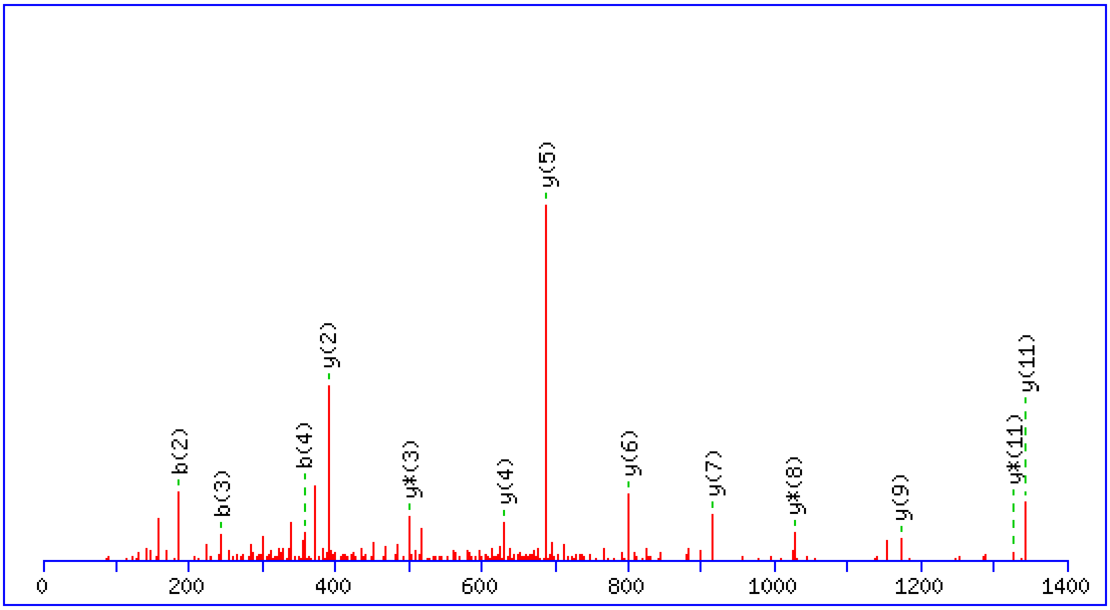

Variable modifications:
Y12 : Phospho (Y)
Ions Score: 61 Expect: 8.2e-05
Matches (Bold Red): 13/124 fragment ions using 18 most intense peaks

#	b	b++	b*	b*++	b0	b0++	Seq.	y	y++	y*	y*++	y0	y0++	#
1	115.05	58.03	98.02	49.52			N							13
2	186.09	93.55	169.06	85.03			A	1415.62	708.31	1398.59	699.80	1397.61	699.31	12
3	243.11	122.06	226.08	113.54			G	1344.58	672.80	1327.56	664.28	1326.57	663.79	11
4	357.15	179.08	340.13	170.57			N	1287.56	644.28	1270.54	635.77	1269.55	635.28	10
5	486.19	243.60	469.17	235.09	468.18	234.60	E	1173.52	587.26	1156.49	578.75	1155.51	578.26	9
6	614.25	307.63	597.23	299.12	596.24	298.62	Q	1044.48	522.74	1027.45	514.23	1026.47	513.74	8
7	729.28	365.14	712.25	356.63	711.27	356.14	D	916.42	458.71	899.39	450.20	898.41	449.71	7
8	842.36	421.69	825.34	413.17	824.35	412.68	L	801.39	401.20	784.36	392.69			6
9	899.39	450.20	882.36	441.68	881.37	441.19	G	688.31	344.66	671.28	336.14			5
10	1012.47	506.74	995.44	498.23	994.46	497.73	I	631.29	316.15	614.26	307.63			4
11	1140.53	570.77	1123.50	562.25	1122.52	561.76	Q	518.20	259.60	501.17	251.09			3
12	1383.56	692.28	1366.53	683.77	1365.55	683.28	Y	390.14	195.57	373.12	187.06			2
13							K	147.11	74.06	130.09	65.55			1

MS2 Spectrum #6. α-catenin pTyr 351

MS/MS Fragmentation of **QALQDLLSEpY^{351}MGNAGR**
Found in **CTNA1_HUMAN**, P35221|CTNA1_HUMAN Catenin alpha-1
Homo sapiens (Human)
Match to Query 650: 1844.753050 from (923.383801,2+)

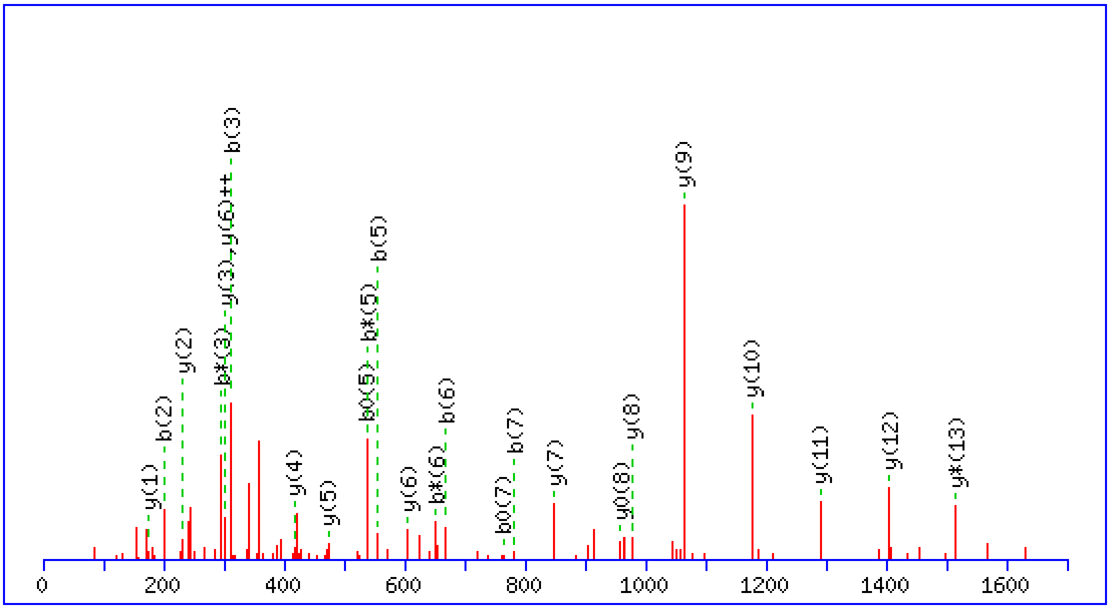

Variable modifications:
Y10 : Phospho (Y)
Ions Score: 63 Expect: 5.5e-05
Matches (Bold Red): 25/158 fragment ions using 66 most intense peaks

#	b	b++	b*	b*++	b0	b0++	Seq.	y	y++	y*	y*++	y0	y0++	#
1	129.07	65.04	112.04	56.52			Q							16
2	200.10	100.56	183.08	92.04			A	1717.76	859.38	1700.73	850.87	1699.75	850.38	15
3	313.19	157.10	296.16	148.58			L	1646.72	823.87	1629.70	815.35	1628.71	814.86	14
4	441.25	221.13	424.22	212.61			Q	1533.64	767.32	1516.61	758.81	1515.63	758.32	13
5	556.27	278.64	539.25	270.13	538.26	269.63	D	1405.58	703.29	1388.56	694.78	1387.57	694.29	12
6	669.36	335.18	652.33	326.67	651.35	326.18	L	1290.55	645.78	1273.53	637.27	1272.54	636.78	11
7	782.44	391.72	765.41	383.21	764.43	382.72	L	1177.47	589.24	1160.44	580.73	1159.46	580.23	10
8	869.47	435.24	852.45	426.73	851.46	426.23	S	1064.39	532.70	1047.36	524.18	1046.38	523.69	9
9	998.52	499.76	981.49	491.25	980.50	490.76	E	977.35	489.18	960.33	480.67	959.34	480.18	8
10	1241.54	621.28	1224.52	612.76	1223.53	612.27	Y	848.31	424.66	831.29	416.15			7
11	1372.59	686.80	1355.56	678.28	1354.57	677.79	M	605.28	303.14	588.26	294.63			6
12	1429.61	715.31	1412.58	706.79	1411.60	706.30	G	474.24	237.62	457.22	229.11			5
13	1543.65	772.33	1526.62	763.82	1525.64	763.32	N	417.22	209.11	400.19	200.60			4
14	1614.69	807.85	1597.66	799.33	1596.68	798.84	A	303.18	152.09	286.15	143.58			3
15	1671.71	836.36	1654.68	827.84	1653.70	827.35	G	232.14	116.57	215.11	108.06			2
16							R	175.12	88.06	158.09	79.55			1

MS2 Spectrum # 7. α-catenin pTyr 563 and 568

MS/MS Fragmentation of **VIHVVTSEMDNpY^{563}EPGVpY^{568}TEK**
Found in **CTNA1_HUMAN**, P35221|CTNA1_HUMAN Catenin alpha-1
Homo sapiens (Human)
Match to Query 718: 2468.949156 from (823.990328,3+)

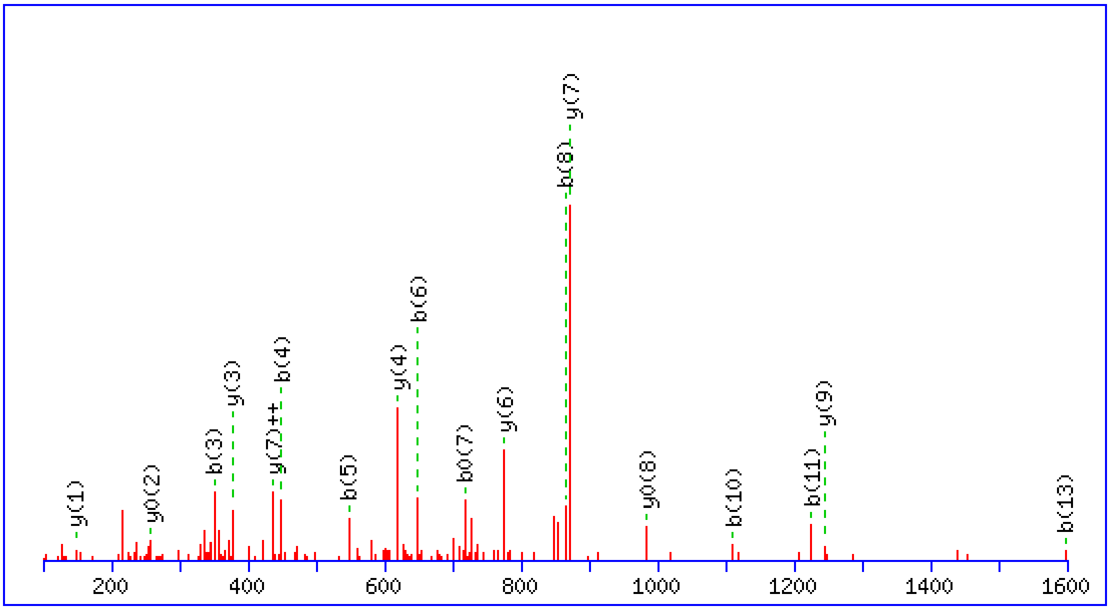

Variable modifications:
Y12 : Phospho (Y)
Y17 : Phospho (Y)
Ions Score: 58 Expect: 5.5e-05
Matches (**Bold Red**): 18/196 fragment ions using 29 most intense peaks

#	b	b++	b*	b*++	b0	b0++	Seq.	y	y++	y*	y*++	y0	y0++	#
1	100.08	50.54					V							20
2	213.16	107.08					I	2370.96	1185.98	2353.93	1177.47	2352.95	1176.98	19
3	350.22	175.61					H	2257.88	1129.44	2240.85	1120.93	2239.87	1120.44	18
4	449.29	225.15					V	2120.82	1060.91	2103.79	1052.40	2102.81	1051.91	17
5	548.36	274.68					V	2021.75	1011.38	2004.72	1002.86	2003.74	1002.37	16
6	649.40	325.21			631.39	316.20	T	1922.68	961.84	1905.65	953.33	1904.67	952.84	15
7	736.44	368.72			718.42	359.72	S	1821.63	911.32	1804.61	902.81	1803.62	902.31	14
8	865.48	433.24			847.47	424.24	E	1734.60	867.80	1717.57	859.29	1716.59	858.80	13
9	996.52	498.76			978.51	489.76	M	1605.56	803.28	1588.53	794.77	1587.55	794.28	12
10	1111.55	556.28			1093.53	547.27	D	1474.52	737.76	1457.49	729.25	1456.51	728.76	11
11	1225.59	613.30	1208.56	604.78	1207.58	604.29	N	1359.49	680.25	1342.46	671.74	1341.48	671.24	10
12	1468.62	734.81	1451.59	726.30	1450.61	725.81	Y	1245.45	623.23	1228.42	614.71	1227.44	614.22	9
13	1597.66	799.33	1580.63	790.82	1579.65	790.33	E	1002.42	501.71	985.39	493.20	984.41	492.71	8
14	1694.71	847.86	1677.69	839.35	1676.70	838.85	P	873.38	437.19	856.35	428.68	855.36	428.19	7
15	1751.73	876.37	1734.71	867.86	1733.72	867.37	G	776.32	388.66	759.30	380.15	758.31	379.66	6
16	1850.80	925.91	1833.78	917.39	1832.79	916.90	V	719.30	360.15	702.27	351.64	701.29	351.15	5
17	2093.83	1047.42	2076.81	1038.91	2075.82	1038.41	Y	620.23	310.62	603.21	302.11	602.22	301.61	4
18	2194.88	1097.94	2177.85	1089.43	2176.87	1088.94	T	377.20	189.11	360.18	180.59	359.19	180.10	3
19	2323.92	1162.47	2306.90	1153.95	2305.91	1153.46	E	276.16	138.58	259.13	130.07	258.14	129.58	2
20							K	147.11	74.06	130.09	65.55			1

MS2 Spectrum # 8. β-catenin pTyr 30

MS/MS Fragmentation of **AAVSHWQQQSpY[30]LDSGIHSGATTTAPSLSGK**
Found in **CTNB1_HUMAN**, P35222|CTNB1_HUMAN Catenin beta-
Homo sapiens (Human)
Match to Query 731: 3164.449527 from (1055.823785,3+)

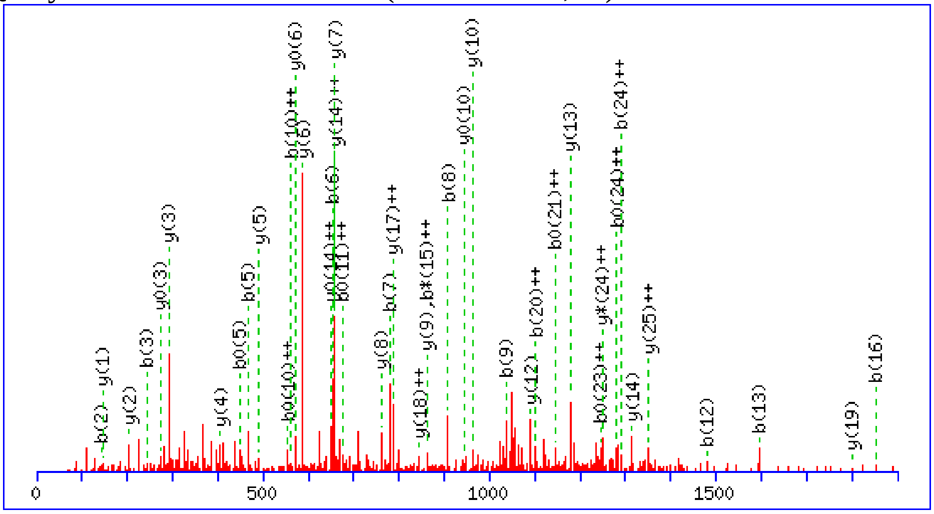

Variable modifications:
Y11 : Phospho (Y)
Ions Score: 62 Expect: 4.8e-05
Matches (Bold Red): 43/326 fragment ions using 109 most intense peaks

#	b	b++	b*	b*++	b0	b0++	Seq.	y	y++	y*	y*++	y0	y0++	#
1	72.04	36.53					A							30
2	143.08	72.04					A	3094.43	1547.72	3077.40	1539.20	3076.42	1538.71	29
3	242.15	121.58					V	3023.39	1512.20	3006.36	1503.69	3005.38	1503.19	28
4	329.18	165.09			311.17	156.09	S	2924.32	1462.66	2907.29	1454.15	2906.31	1453.66	27
5	466.24	233.62			448.23	224.62	H	2837.29	1419.15	2820.26	1410.63	2819.28	1410.14	26
6	652.32	326.66			634.31	317.66	W	2700.23	1350.62	2683.20	1342.11	2682.22	1341.61	25
7	780.38	390.69	763.35	382.18	762.37	381.69	Q	2514.15	1257.58	2497.12	1249.07	2496.14	1248.57	24
8	908.44	454.72	891.41	446.21	890.43	445.72	Q	2386.09	1193.55	2369.07	1185.04	2368.08	1184.54	23
9	1036.50	518.75	1019.47	510.24	1018.49	509.75	Q	2258.03	1129.52	2241.01	1121.01	2240.02	1120.52	22
10	1123.53	562.27	1106.50	553.75	1105.52	553.26	S	2129.98	1065.49	2112.95	1056.98	2111.96	1056.49	21
11	1366.56	683.78	1349.53	675.27	1348.55	674.78	Y	2042.94	1021.98	2025.92	1013.46	2024.93	1012.97	20
12	1479.64	740.32	1462.62	731.81	1461.63	731.32	L	1799.91	900.46	1782.89	891.95	1781.90	891.46	19
13	1594.67	797.84	1577.64	789.32	1576.66	788.83	D	1686.83	843.92	1669.80	835.41	1668.82	834.91	18
14	1681.70	841.35	1664.67	832.84	1663.69	832.35	S	1571.80	786.40	1554.78	777.89	1553.79	777.40	17
15	1738.72	869.86	1721.70	861.35	1720.71	860.86	G	1484.77	742.89	1467.74	734.38	1466.76	733.88	16
16	1851.81	926.41	1834.78	917.89	1833.80	917.40	I	1427.75	714.38	1410.72	705.86	1409.74	705.37	15
17	1988.87	994.94	1971.84	986.42	1970.85	985.93	H	1314.66	657.84	1297.64	649.32	1296.65	648.83	14
18	2075.90	1038.45	2058.87	1029.94	2057.89	1029.45	S	1177.61	589.31	1160.58	580.79	1159.60	580.30	13
19	2132.92	1066.96	2115.89	1058.45	2114.91	1057.96	G	1090.57	545.79	1073.55	537.28	1072.56	536.79	12
20	2203.96	1102.48	2186.93	1093.97	2185.95	1093.48	A	1033.55	517.28	1016.53	508.77	1015.54	508.27	11
21	2305.00	1153.01	2287.98	1144.49	2286.99	1144.00	T	962.52	481.76	945.49	473.25	944.50	472.76	10
22	2406.05	1203.53	2389.02	1195.02	2388.04	1194.52	T	861.47	431.24	844.44	422.72	843.46	422.23	9
23	2507.10	1254.05	2490.07	1245.54	2489.09	1245.05	T	760.42	380.71	743.39	372.20	742.41	371.71	8
24	2578.14	1289.57	2561.11	1281.06	2560.13	1280.57	A	659.37	330.19	642.35	321.68	641.36	321.18	7
25	2675.19	1338.10	2658.16	1329.58	2657.18	1329.09	P	588.34	294.67	571.31	286.16	570.32	285.67	6
26	2762.22	1381.61	2745.19	1373.10	2744.21	1372.61	S	491.28	246.14	474.26	237.63	473.27	237.14	5
27	2875.30	1438.16	2858.28	1429.64	2857.29	1429.15	L	404.25	202.63	387.22	194.12	386.24	193.62	4
28	2962.34	1481.67	2945.31	1473.16	2944.33	1472.67	S	291.17	146.09	274.14	137.57	273.16	137.08	3
29	3019.36	1510.18	3002.33	1501.67	3001.35	1501.18	G	204.13	102.57	187.11	94.06			2
30							K	147.11	74.06	130.09	65.55			1

Supplementary Figure S1b

Tyrosine phosphorylated E-Cdh and Ctn peptides used for the immunization to generate antibodies that specifically recognize these phosphorylated variants

E-Cdh (Y753/754)

Mix of 2 different phospho-peptides

DTRDNV[P]YYYDEEG

DTRDNVY[P]YYDEEG

E-Cdh (Y859)

CSDKDQDY[P]DYLNE

E-Cdh (Y876)

KKLADMY[P]GGGEDD

α-Ctn (Y177)

CDLGIQY[P]KALKPE

α-Ctn (Y351)

QDLLSEY[P]MGNAGR

α-Ctn (Y563/568)

Mix of 2 different phospho-peptides

CMDNYEPGVY[P]TEK

CMDNY[P]EPGVYTEK

β-Ctn (Y30)

HWQQQSY[P]LDSGIH

Supplementary Figure S2

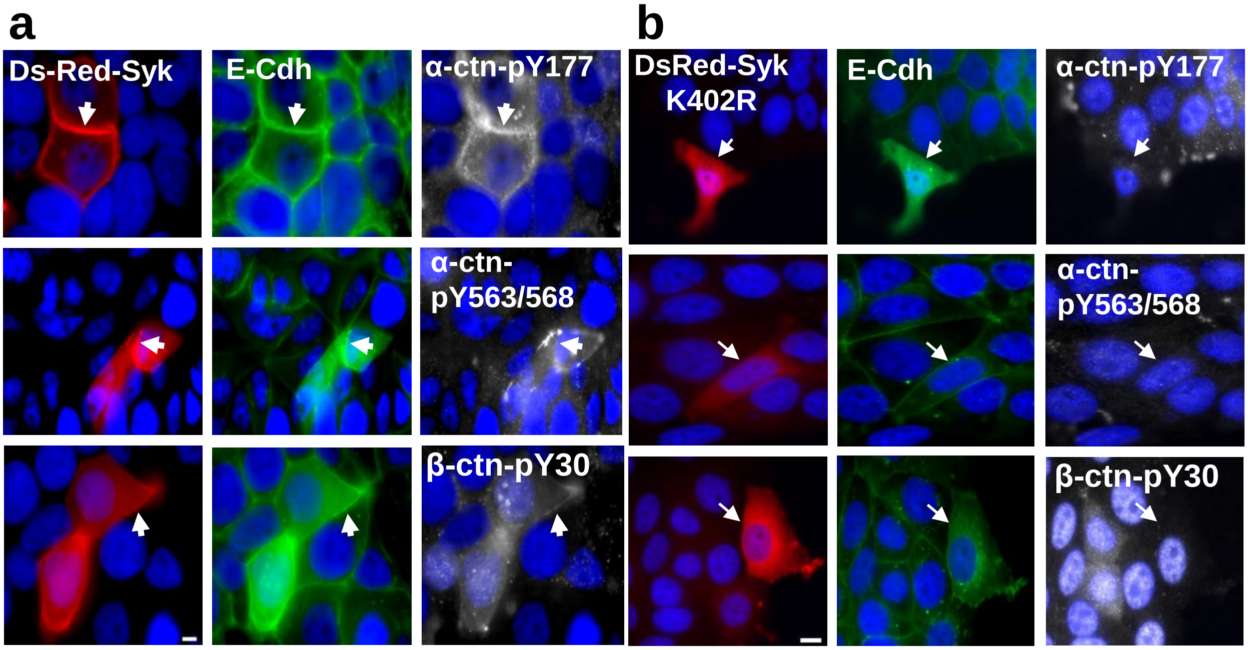

Supplem. Fig. S2 Catenin phosphorylation is induced by catalytically active DsRed-Syk expression.
MCF7 cells expressing WT (**a**) or kinase-dead/ K402R (**b**) DsRed-Syk. Tyrosine phosphorylation of α- and β-Ctn (Cy5/white) and DNA (Hoechst/blue) were assessed as in Fig. 1d-e. Thick arrows: co-localization of endogenous E-Cdh with phosphorylated E-Cdh/α-Ctn at adherens junctions. Thin arrows: E-Cdh-positive cell junctions (FITC/green) in kinase-dead DsRed-Syk-positive cells, but without α-Ctn or β-Ctn phosphorylation at the indicated tyrosine residues. Scale bar: 10 μm.

Supplementary Figure S3

a

Quantification of E-Cdh signal
(px intensity) at intercellular junctions

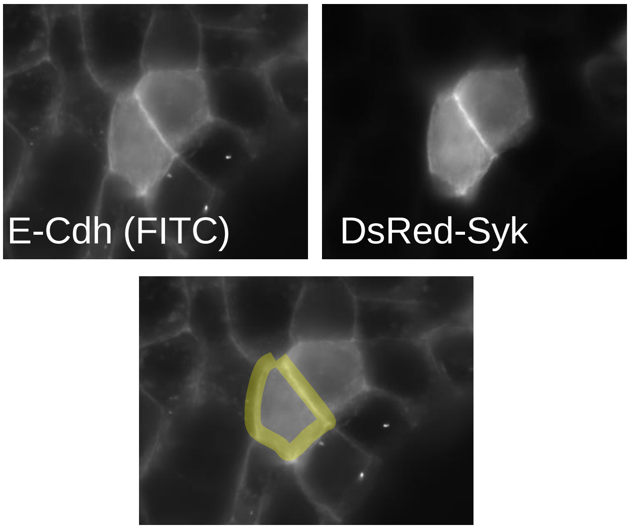

b

Colocalization profile intensity

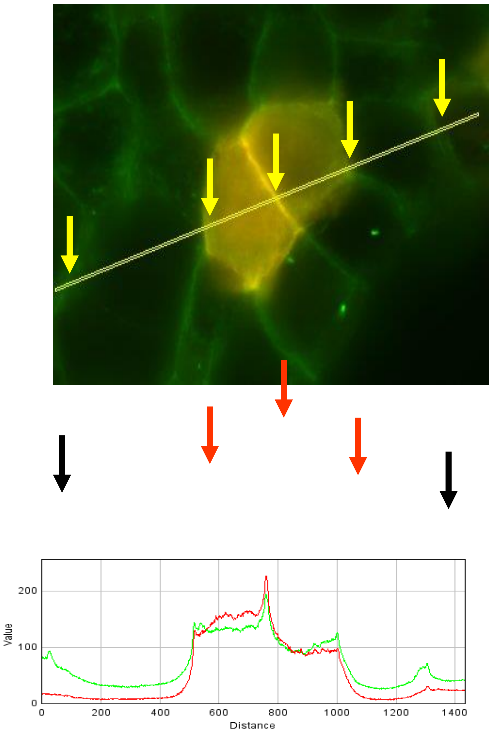

Supplem. Fig S3: Transient DsRed-Syk transfection increases E-Cdh signal intensity at intercellular junctions.
(**a**) Quantification of the E-Cdh signal (pix intensity) at intercellular junctions in MCF7 cells expressing (right) or not (left) DsRed-Syk shows a 1.99±0.38-fold increase of this signal at intercellular junctions of transfected cells ($p<0.001$; paired t-test). (**b**) Co-localization profile of E-Cdh (FITC/green) and Syk (TRITC/red) in DsRed-Syk cells using ImageJ.

Supplementary Figure S4

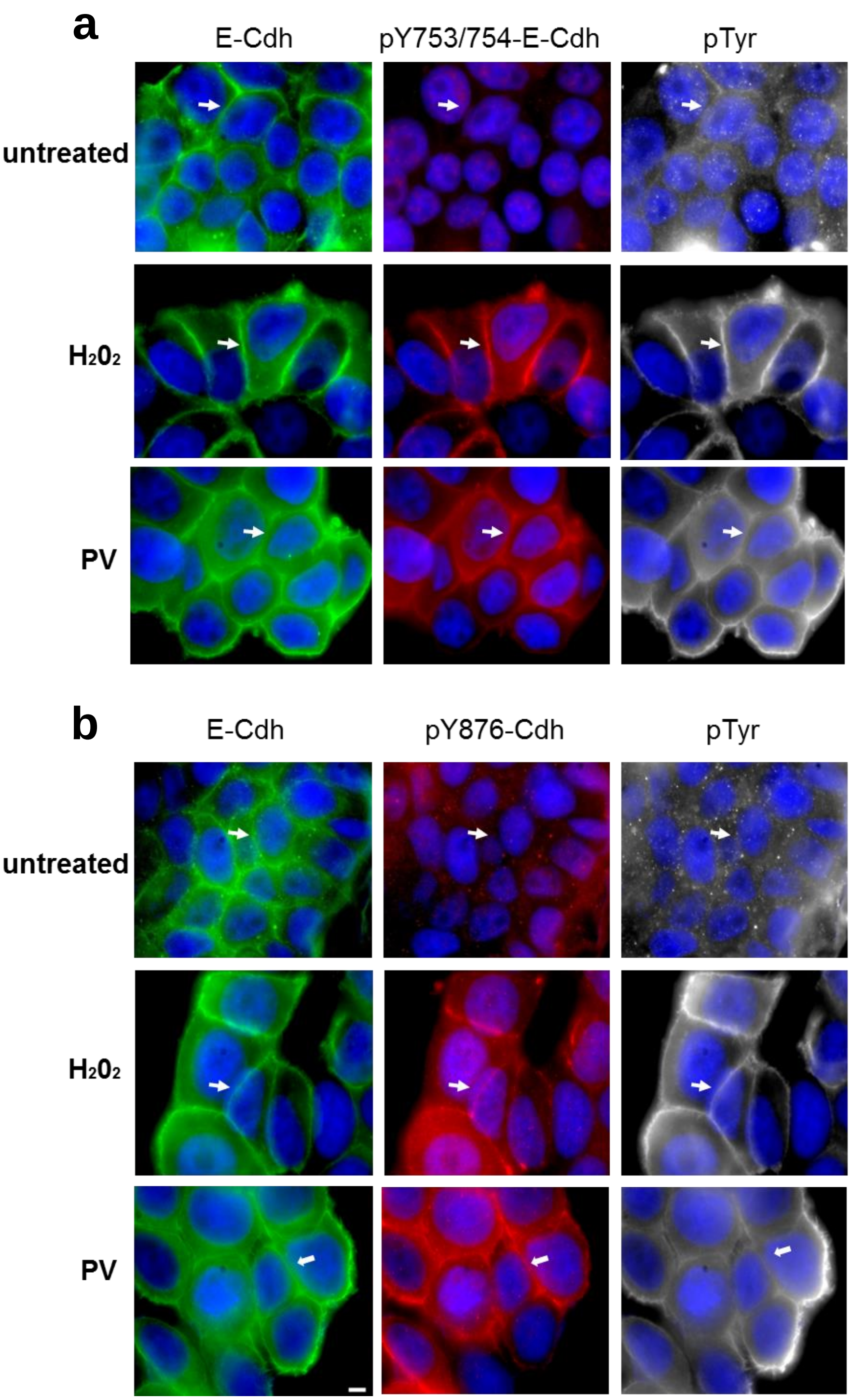

Supplem. Fig. S4: Oxidative stress increases E-Cdh phosphorylation at adherens junctions.
MCF7 cells were incubated with H2O2 (10 mM) or sodium peroxyvanadate (PV, 1 mM). Anti-E-Cdh-FITC (green) was used to detect adherens junctions. Phosphorylation was detected using the anti-pY-E-Cdh antibodies (TRITC/red) and a generic pTyr antibody (Cy5/white) and DNA with Hoechst (blue). Arrows indicate the co-localization of total E-Cdh and phosphorylated E-Cdh in treated cells. Scale bar: 10 µm.

Supplementary Figure S5

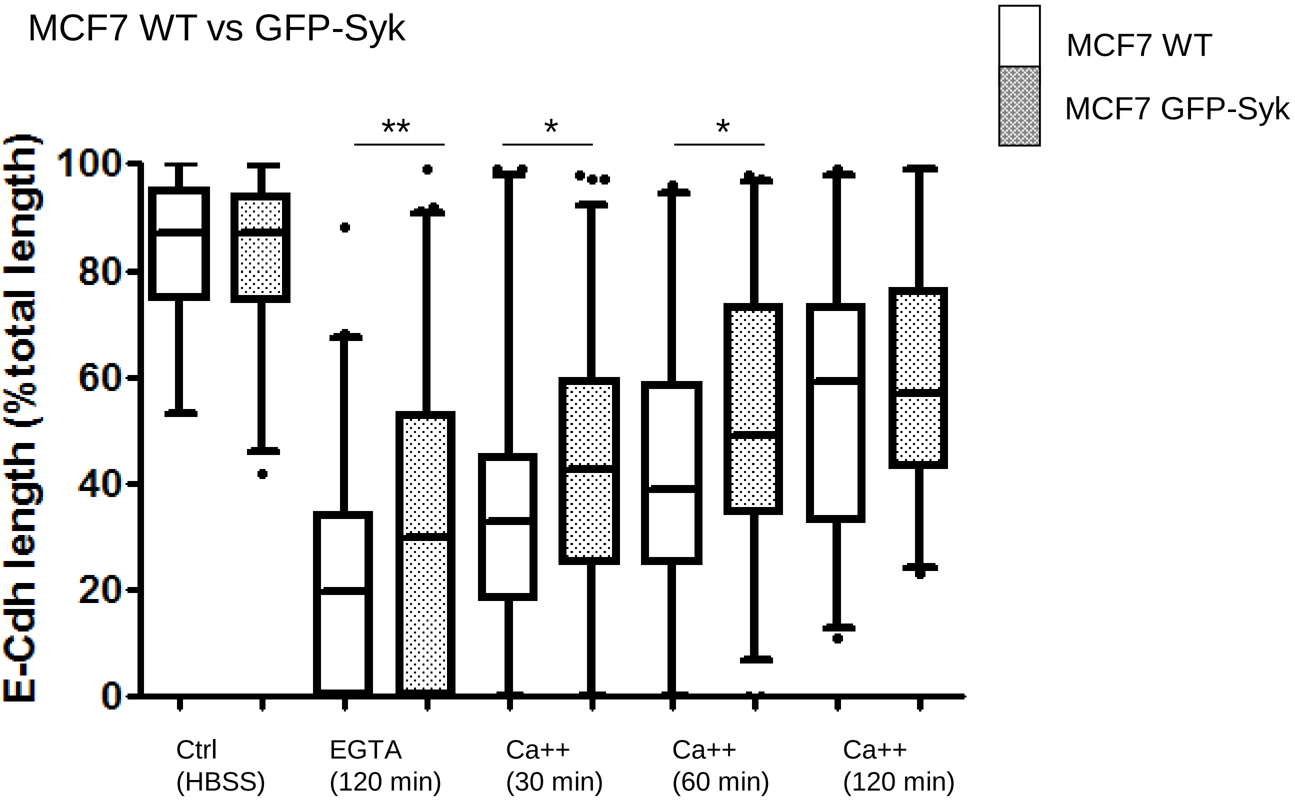

Supplem. Fig. S5: GFP-Syk expression promotes 2D cell aggregation.
2D-cell re-aggregation assays using parental (WT) and GFP-Syk MCF7 cells. Box-and-whiskers plot showing the percentage of E-Cdh-positive border length relative to the total cell border at the indicated time points; ** p <0.01, *p <0.05 (paired t-test).

Supplementary Figure S6

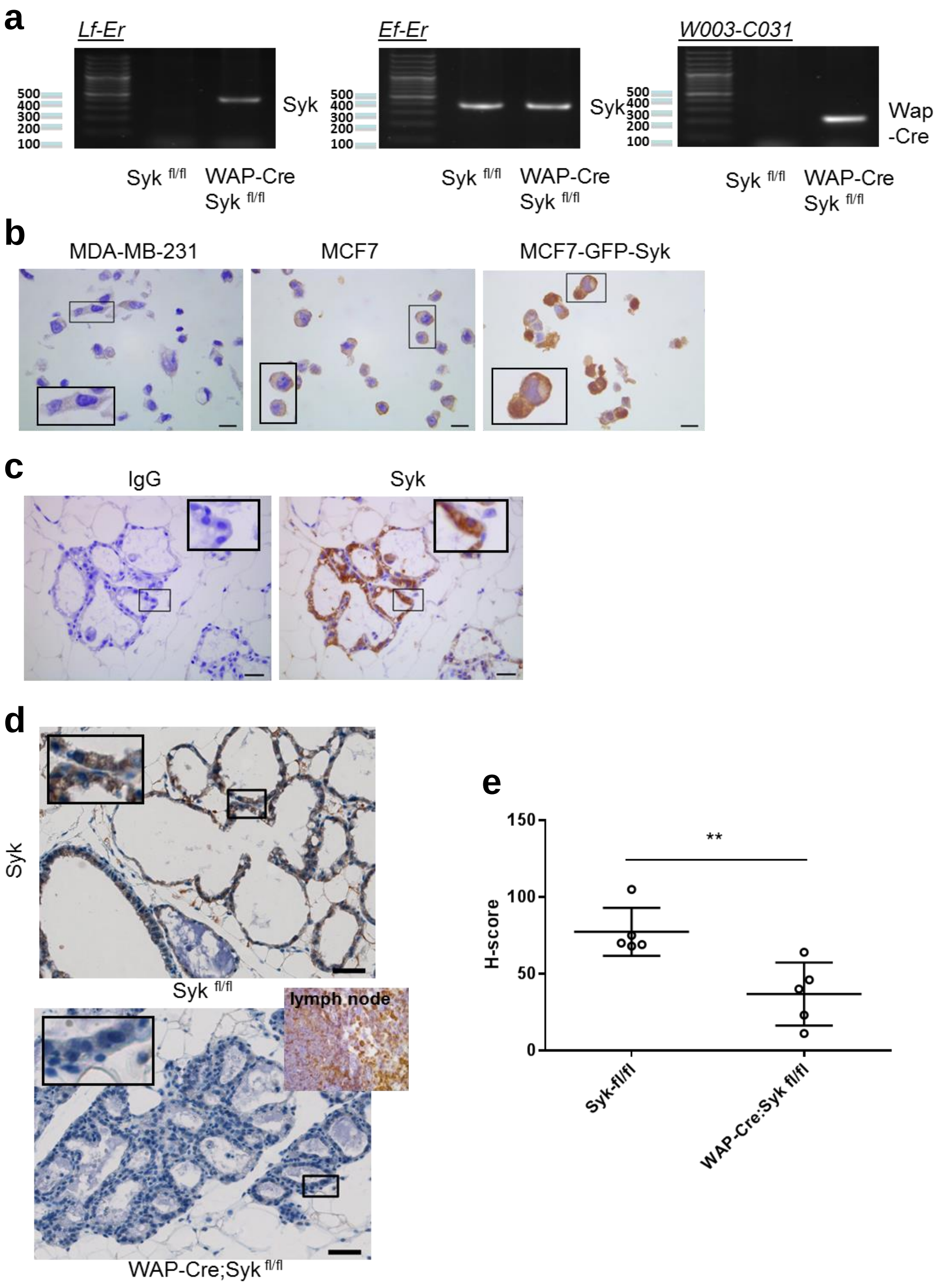

Supplem. Fig S6: Validation of Syk knockout in Wap-Cre;Syk$^{fl/fl}$ mice.
(a) Mammary epithelial cells from weaned mice were analyzed by primers detecting the excised (442bp) or floxed (399 bp) Syk locus and Wap-Cre (210 bp). The anti-Syk antibody specificity was validated by immunohistochemical analysis of (**b**) paraffin-embedded breast cancer cells, and (**c**) paraffin-embedded inguinal mammary gland sections of Sykfl/fl mice at weaning; IgG, non-specific serum. (**d**) Immunohistochemical Syk analysis in Sykfl/fl and Wap-Cre;Sykfl/fl inguinal mammary gland tissue sections at weaning. Lymph node sections were used as positive control for Syk expression. (**e**) Quantification of cytoplasmic Syk expression (H score) in inguinal mammary gland sections at weaning (n=5/group; mean ± SEM; ** p <0.01, Mann-Whitney test). Scale bars: 10 µm.

Supplementary Figure S7

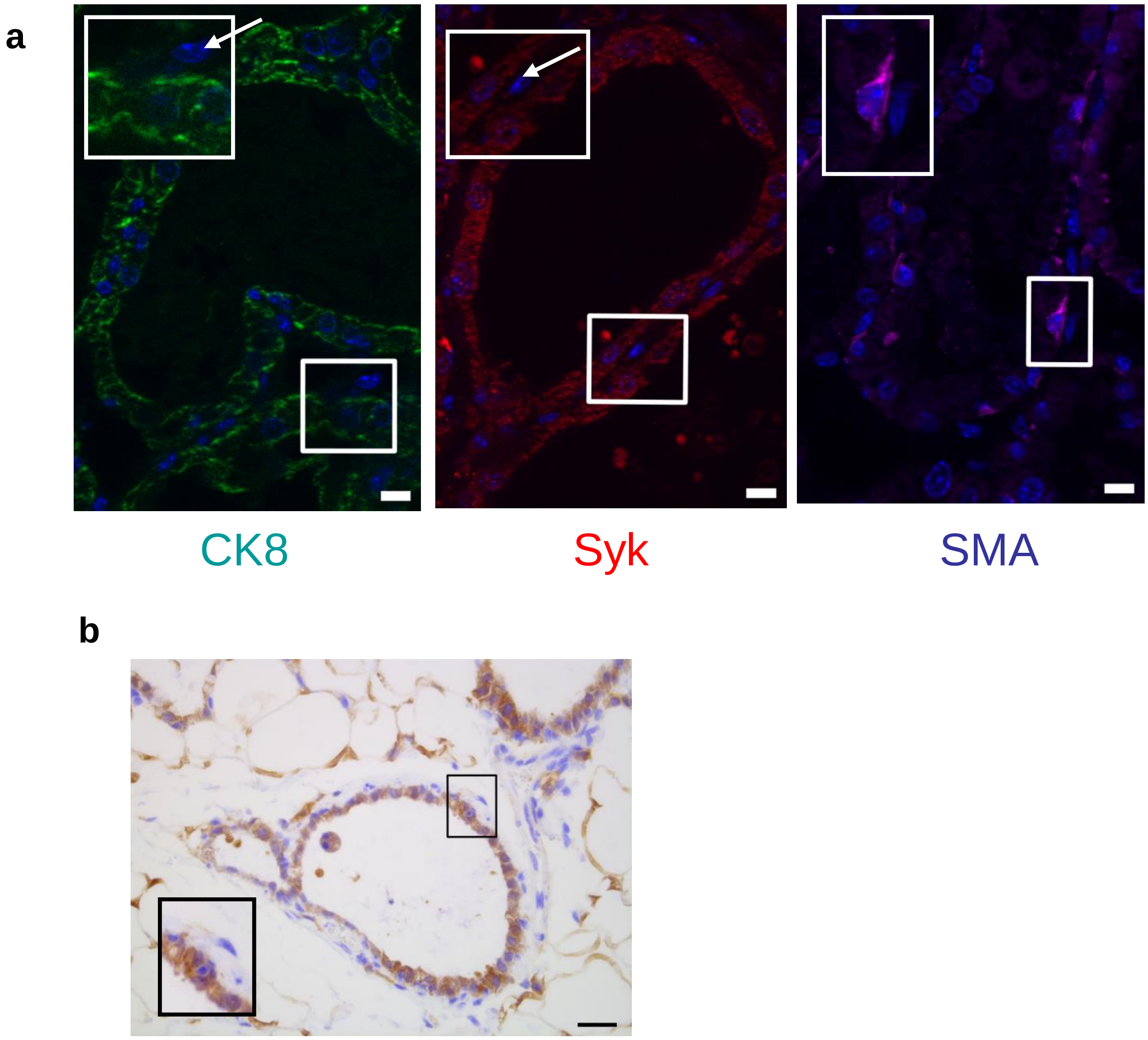

Supplem. Fig. S7: Syk is expressed in luminal epithelial cells of the mammary gland. (**a**) Paraffin-embedded mammary gland tissue sections from *Syk*$^{fl/fl}$ ^{mice} at weaning were immunostained with antibodies against anti-Syk (TRITC/red), anti-cytokeratin-8 (CK8, a luminal marker, GFP/green), and anti-SMA (myoepithelial/basal marker; Cy5/purple). DNA was stained with Hoechst (blue). Arrows indicate a Syk- and CK8-negative myo-epithelial cell. (**b**) Syk expression in paraffin-embedded *Syk*$^{fl/fl}$ mammary gland tissue sections. Insets show enlarged Syk-negative myoepithelial cells versus Syk-positive luminal epithelial cells. Scale bars: 10 μm.

Supplementary Figure S8

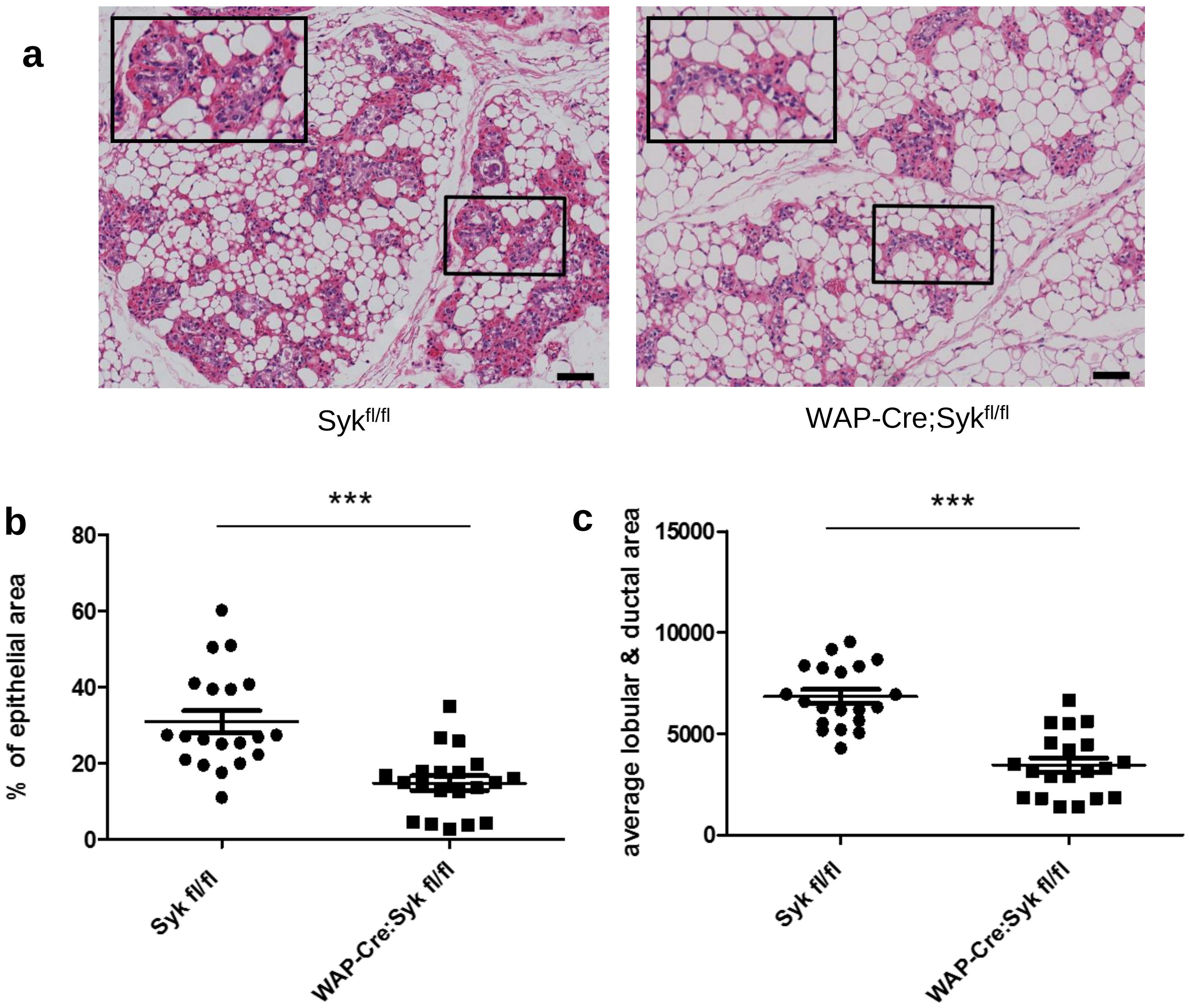

Supplem. Fig. S8: Syk loss negatively affects epithelial morphogenesis in involuting mammary glands.
(**a**) Hematoxylin/eosin staining of involuting inguinal mammary glands from Sykfl/fl and Wap-Cre;Sykfl/fl mice at day 3 post-weaning. Scale bar: 10 µm (**b**) Percentage of mammary epithelium (ducts and lobules) relative to the total area in each studied field (epithelium and stroma). (**c**) Mean area of mammary epithelium (ducts and lobules) in each studied field in pix2 (n= 4 for Sykfl/fl and Wap-Cre;Sykfl/fl, 5 fields measured for each mouse; mean ± SEM; *** p <0.001, t-test).
